# Association Between Preoperative Obstructive Sleep Apnea and Preoperative Positive Airway Pressure With Postoperative Intensive Care Unit Delirium

**DOI:** 10.1001/jamanetworkopen.2020.3125

**Published:** 2020-04-20

**Authors:** Christopher R. King, Bradley A. Fritz, Krisztina Escallier, Yo-El S. Ju, Nan Lin, Sherry McKinnon, Michael S. Avidan, Ben Julian Palanca

**Affiliations:** 1 Department of Anesthesiology, Washington University in St Louis, St Louis, Missouri; 2 Department of Anesthesiology and Perioperative Medicine, University of California Los Angeles; 3 Department of Neurology, Washington University in St Louis, St Louis, Missouri; 4 Department of Mathematics and Statistics, Washington University in St Louis, St Louis, Missouri

## Abstract

**Importance:**

Obstructive sleep apnea has been associated with postoperative delirium, which predisposes patients to major adverse outcomes. Positive airway pressure may be an effective intervention to reduce delirium in this population.

**Objectives:**

To determine if preoperative obstructive sleep apnea is associated with postoperative incident delirium in the intensive care unit and if preoperative positive airway pressure adherence modifies the association.

**Design, Setting, and Participants:**

A retrospective single-center cohort study was conducted at a US tertiary hospital from November 1, 2012, to August 31, 2016, among 7792 patients admitted to an intensive care unit who underwent routine Confusion Assessment Method for the intensive care unit after major surgery. Patients were adults who had undergone a complete preoperative anesthesia assessment, received general anesthesia, underwent at least 1 delirium assessment, were not delirious preoperatively, and had a preoperative intensive care unit stay of less than 6 days. Statistical analysis was conducted from August 20, 2019, to January 11, 2020.

**Exposures:**

Self-reported obstructive sleep apnea, billing diagnosis of obstructive sleep apnea, or STOP-BANG (Snoring, Tiredness, Observed Apnea, Blood Pressure, Body Mass Index, Age, Neck Circumference and Gender) questionnaire score greater than 4, as well as self-reported use of preoperative positive airway pressure.

**Main Outcomes and Measures:**

Delirium within 7 days of surgery.

**Results:**

A total of 7792 patients (4562 men; mean [SD] age, 59.2 [15.3] years) met inclusion criteria. Diagnosed or likely obstructive sleep apnea occurred in 2044 patients (26%), and delirium occurred in 3637 patients (47%). The proportion of patients with incident delirium was lower among those with obstructive sleep apnea than those without (897 of 2044 [44%] vs 2740 of 5748 [48%]; unadjusted risk difference, −0.04; 99% credible interval [CrI], −0.07 to −0.00). Positive airway pressure adherence had minimal association with delirium (risk difference, −0.00; 99% CrI, −0.09 to 0.09). Doubly robust confounder adjustment eliminated the association between obstructive sleep apnea and delirium (risk difference, −0.01; 99% CrI, −0.04 to 0.03) and did not change that of preoperative positive airway pressure adherence (risk difference, −0.00, 99% CrI, −0.07 to 0.07). The results were consistent across multiple sensitivity analyses.

**Conclusions and Relevance:**

After risk adjustment, this study found no association between obstructive sleep apnea and postoperative delirium in the context of usual care in the intensive care unit, with 99% CrIs excluding clinically meaningful associations. With limited precision, no association was found between positive airway pressure adherence and delirium. Selection bias and measurement error limit the validity and generalizability of these observational associations; however, they suggest that interventions targeting sleep apnea and positive airway pressure are unlikely to have a meaningful association with postoperative intensive care unit delirium.

## Key Points

**Question:**

Is there an association between obstructive sleep apnea and delirium after major surgery?

**Findings:**

In this cohort study of 7792 patients admitted to the intensive care unit after surgery, 26% had obstructive sleep apnea, and delirium occurred in 47%. After risk adjustment, there was no significant association between obstructive sleep apnea and postoperative delirium.

**Meaning:**

This study found no association between obstructive sleep apnea and delirium in patients admitted postoperatively to the intensive care unit.

## Teaser

This cohort study examines whether preoperative obstructive sleep apnea is associated with postoperative incident delirium in the intensive care unit (ICU) and if preoperative positive airway pressure adherence modifies the association.

## Introduction

Postoperative delirium is a common^1,2^ and serious complication associated with increased mortality,^3^ prolonged intensive care unit (ICU) stays,^4^ and decreased quality of life.^5^ Several groups have identified obstructive sleep apnea (OSA) as a risk factor for postoperative delirium with a strong effect size.^6,7,8,9,10^ A recent prospective study identified severe undiagnosed OSA as a substantial risk factor for postoperative cardiac complications but found limited association between OSA and postoperative delirium.^11^ There is biological plausibility for an association between OSA and delirium; OSA likely causes hypoxia, inflammation, and disrupted sleep architecture, which are likely associated with delirium.^12,13,14^ Positive airway pressure (PAP) improves OSA symptoms^15^ and has been suggested to mitigate these potential mediators but does not seem to strongly affect cardiovascular outcomes.^16,17^ A randomized intervention of postoperative PAP had mixed results,^9^ and some large observational studies found limited association between OSA and postoperative mortality.^18^

Our goal was to examine the associations between OSA, preoperative PAP adherence, and postoperative delirium in a large, diverse cohort of patients undergoing major surgery with ICU admission. We had 2 coprimary hypotheses: that patients with diagnosed or likely OSA would have an increased incidence of postoperative delirium and that patients adherent to PAP therapy for OSA would have a reduced incidence of postoperative delirium.

## Methods

The protocol for the current study contains additional details on databases, power calculations, and analytic choices.^19^ In brief, this is a single-center retrospective cohort study of patients who underwent major surgery from November 1, 2012, to August 31, 2016. Inclusion criteria were completion of a preoperative evaluation, aged 18 years or older, receipt of general anesthesia, admission to an ICU routinely performing the Confusion Assessment Method for the ICU (CAM-ICU), documentation of at least 1 CAM-ICU, and no positive CAM-ICU result during the preceding 72 hours. Departing from our analysis plan (discussed in more detail in eAppendix 3 and eFigure 2 in the Supplement), we also excluded patients with long (≥6 days) preceding ICU stays owing to concerns about selection bias. The exclusion of some ICUs is also a protocol departure and functionally excludes patients undergoing neurosurgical procedures and those in medical ICUs. Records for nonanalyzed patients were also obtained for auxiliary uses (eAppendix 3 and eFigure 2 in the Supplement). The Human Research Protection Office at Washington University School of Medicine in St Louis approved this study with a waiver of consent, as the project presents minimal risk to patients and a deidentified data set was created for analysis. High-resolution clinical histories linked to administrative records make data reidentification a serious risk; therefore, this secondary data set is available by institutional review board application only. This study followed the Strengthening the Reporting of Observational Studies in Epidemiology (STROBE) reporting guideline.

The preoperative evaluations included medical and surgical history, self-reported OSA, and self-reported adherence to PAP therapy among those reporting an OSA diagnosis. After April 2014, the STOP-BANG (Snoring, Tiredness, Observed Apnea, Blood Pressure, Body Mass Index, Age, Neck Circumference and Gender) screening—a validated questionnaire combining symptoms, comorbidities, anthropometric characteristics, and demographic characteristics that are associated with OSA^20,21^—was conducted in our preoperative clinic. We linked preoperative evaluations to administrative data and electronic health records. We extracted CAM-ICU assessments for the initial postoperative ICU stay, which were routinely performed twice daily by ICU nurses for patients with adequate mental status (Richmond Agitation-Sedation Scale score >–4 on a scale of –5 to 4, where –5 indicates unarousable sedation and 4 indicates combative behavior). We extracted demographic variables and the contact address from administrative records. Addresses were linked to 2010 census zip code tabulation area socioeconomic variables, including those from the American Community Survey.^22^ Procedure billing codes were mapped to the Agency for Healthcare Research and Quality Clinical Classification Scheme^23^ to create approximately homogeneous groups of surgical morbidity and to the risk stratification index^24^ log hazard ratio for 1-year mortality to calibrate the morbidity of each procedure.

The primary exposure was defined as either a clinician-noted OSA diagnosis, billing diagnosis of OSA, or screening results indicating the patient was at high risk for OSA (STOP-BANG score, >4 on a scale of 0 to 8, where a higher score indicates greater risk). The secondary exposure was self-reported preoperative PAP adherence among patients with OSA. Because most patients with diagnosed OSA reported having a prescription for PAP therapy and either routine use or nonadherence, this exposure was dichotomized as some PAP adherence vs none. Patients who used bilevel PAP therapy for respiratory failure without diagnosed OSA were not included. Additional data cleaning and linking details are contained in eAppendix 1 in the Supplement. Clinician OSA diagnosis was never considered missing; the field was populated with “no” by default without information on whether the question was answered.

### Statistical Analysis

Statistical analysis was conducted from August 20, 2019, to January 11, 2020. All statistical tests were 2-sided. Details of missing data imputation methods and a detailed exploration of missing outcomes is contained in eAppendix 2, eAppendix 3, eTable 2, eTable 3, eFigure 2, eFigure 3, and eFigure 4 in the Supplement. Treatment effects were estimated using an approach based on Bayesian additive regression trees^25^ titled Bayesian Causal Forests.^26^ Briefly, this method first estimates a propensity score using nonparametric regression, then uses nonparametric regression to estimate the outcome surface in the exposed group and the controls using propensity scores and covariates. The response surface in the exposed group is “shrunk” toward that of the controls, but trees unique to the exposed group allow heterogeneity of the treatment effect. Details of the analysis are in eAppendix 4, eAppendix 6, and eFigure 1 in the Supplement, and multiple sensitivity analyses are in eAppendix 5 and eAppendix 7 in the Supplement. eTable 6 in the Supplement reports effect sizes when giving all patients who were not assessed by the CAM-ICU a positive result, giving all patients who were not assessed by the CAM-ICU a negative result, and reporting imputed outcomes based on their baseline characteristics. We include matched non-ICU patients with similar procedures and baseline characteristics as negative for delirium. We restricted the sample to several subsets with plausibly higher data quality. We also computed propensity scores and estimated treatment effects by alternative algorithms. We experimented with restricted sets of confounders and excluded STOP-BANG from the exposure.

In our protocol, we anticipated mediation analyses using intraoperative variables and postoperative medications. However, we believed that, given the null overall findings, a mediation analysis was unlikely to be clinically meaningful. We deemed that stratification into hypoactive and hyperactive delirium was no longer meaningful based on the findings below, and such stratification is not reported. That is, the primary analysis was used as a gatekeeper to avoid forking-paths multiplicity that contributes to false-positive reports.^27^ A sensitivity analysis including potential mediators is included in eTable 6 in the Supplement; this corresponds roughly to the “direct” effect of OSA, but we treat it as exploratory.

Calculations were performed in R, version 3.5.1.^28^ Source code (without data) is available at https://github.com/cryanking/osa_delirium_wusm. We present 99% CIs and the bayesian analogue, credible intervals (CrIs).

## Results

**Figure 1.**
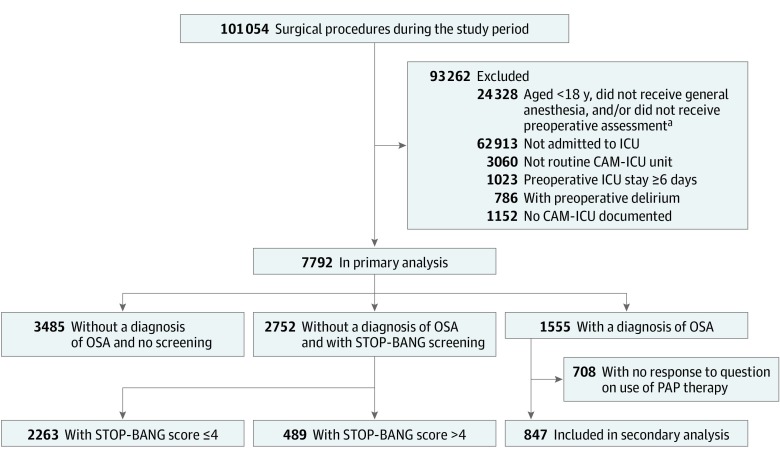
Participant Flow Diagram Patients with a billing diagnosis of obstructive sleep apnea (OSA) are included in “OSA diagnosis.” See eAppendix 3, eFigure 2, and eTable 2 in the Supplement for “Not routine CAM-ICU unit” (an intensive care unit [ICU] performing the Confusion Assessment Method for the ICU [CAM-ICU] on f<70% of patients). PAP indicates positive airway pressure; and STOP-BANG, Snoring, Tiredness, Observed Apnea, Blood Pressure, Body Mass Index, Age, Neck Circumference and Gender. ^a^Overlapping categories.

Figure 1 provides a flow diagram of the included patients. A total of 7792 patients (4562 men [59%]; 6135 white race [79%]; median American Society of Anesthesiologists physical status, 3 [interquartile range, 3-4]; mean [SD] age, 59.2 [15.3] years) met inclusion criteria (Table 1; eTable 1 in the Supplement). Diagnosed OSA was common in the analytic population (1555 [20%]), of whom 708 (46%) responded to the PAP adherence question. After April 2014, 92% of all surgical patients (42 355 of 45 877) responded to the STOP-BANG questionnaire; among the analytic cohort without OSA, the fraction who underwent STOP-BANG screening was 79% (3701 of 4666). Of the 847 patients who responded to the PAP therapy question, 511 (60%) reported routine adherence. Means, covariances, and association of STOP-BANG elements with postoperative delirium are presented in eTable 4 in the Supplement; eTable 5 in the Supplement contains missingness rates. Among those without an OSA diagnosis who were screened by STOP-BANG, 489 of 2275 (21%) scored above 4. Considering diagnosis and screening together, compared with patients in the ICU without OSA, those with OSA had higher rates of cardiac surgery (970 of 2044 [48%] vs 2522 of 5748 [44%]) and greater overall comorbidity (median Charlson Comorbidity Index, 3 [interquartile range, 2-5] vs 2 [interquartile range, 1-4]) (Table 1).

**Table 1.** Association of Baseline Factors With OSA in Analytic Cohort^a^

Characteristic	No OSA (n = 5748)	OSA (n = 2044)	Effect size (95% CI)^b^	*P* value^c^
Female sex, No. (%)	2560 (45)	670 (33)	0.24 (0.19 to 0.29)	<.001
Age, mean (SD), y	58.3 (16.2)	61.7 (11.9)	−0.22 (−0.27 to 0.17)	<.001
Race/ethnicity, No. (%)				
Unknown	174 (3)	41 (2)	0.08 (0.06 to 0.10)	<.001
Black	905 (16)	250 (12)		
White	4421 (77)	1714 (84)		
Other	248 (4)	39 (2)		
Surgery group, No. (%)^d^				
Other organ transplant	390 (7)	88 (4)	0.07 (0.04 to 0.08)	<.001
Cardiovascular system	2522 (44)	970 (48)		
Digestive system	1026 (18)	311 (15)		
Female genital organs	65 (1)	21 (1)		
Integumentary system	81 (1)	32 (2)		
Musculoskeletal system	739 (13)	315 (15)		
Nervous system	123 (2)	33 (3)		
Respiratory system	255 (4)	75 (4)		
Urinary system	165 (3)	58 (3)		
Other	382 (7)	141 (7)		
ASA physical status, No. (%)				
1	67 (1)	4 (0.2)	0.11 (0.08 to 0.13)	<.001
2	814 (14)	182 (9)		
3	2229 (39)	920 (45)		
4	2492 (43)	922 (45)		
5	146 (3)	16 (1)		
CAD, No. (%)	1456 (25)	787 (39)	−0.29 (−0.34 to 0.24)	<.001
Atrial fibrillation, No. (%)	417 (7)	284 (14)	−0.23 (−0.28 to 0.18)	<.001
COPD, No. (%)	685 (12)	422 (21)	−0.25 (−0.30 to 0.20)	<.001
CKD, No. (%)	750 (13)	473 (23)	−0.28 (−0.33 to 0.23)	<.001
Dementia, No. (%)	30 (1)	13 (1)	−0.02 (−0.07 to 0.04)	.57
Hypertension, No. (%)	2514 (44)	1455 (71)	−0.57 (−0.62 to 0.51)	<.001
BMI, mean (SD)	27.5 (6.5)	33.5 (8.4)	−0.84 (−0.89 to 0.78)	<.001
BMI missing, No. (%)	611 (11)	55 (3)	0.29 (0.24 to 0.34)	<.001
CCI, median (IQR)	2 (1-4)	3 (2-5)	−0.23 (−0.28 to 0.18)	<.001
CCI missing, No. (%)	61 (1)	18 (1)	0.02 (−0.03 to 0.07)	.47
Risk index, mean (SD), log HR	−0.44 (0.47)	−0.47 (0.48)	0.05 (0.00 to 10.0)	.05
Risk index missing, No. (%)	211 (4)	80 (4)	−0.01 (−0.06 to 0.04)	.65
ZCTA poverty, mean (SD), %^e^	16 (9.7)	16 (9.0)	0.04 (−0.02 to 0.10)	.15
ZCTA missing, No. (%)	1865 (32)	652 (32)	0.01 (−0.04 to 0.06)	.65
Postoperative benzodiazepine order, No. (%)	1611 (28)	544 (27)	0.03 (−0.02 to 0.08)	.22
Postoperative sedation, No. (%)^f^	1349 (24)	455 (22)	0.03 (−0.02 to 0.08)	.26
Postoperative ventilation, No. (%)	3474 (60)	1320 (65)	−0.09 (−0.14 to 0.03)	<.001
Postoperative NI-PAP, No. (%)	1055 (18)	750 (37)	−0.44 (−0.49 to 0.39)	<.001
Intraoperative OME, mean (SD), mg^g^	90 (61)	98 (63)	−0.13 (−0.18 to 0.08)	<.001
Midazolam dose, mean (SD), mg	2.6 (3.0)	2.5 (2.9)	0.03 (−0.02 to 0.08)	.19

Abbreviations: ASA, American Society of Anesthesiologists; BMI, body mass index (calculated as weight in kilograms divided by height in meters squared); CAD, coronary artery disease; CCI, Charlson Comorbidity Index; CKD, chronic kidney disease; COPD, chronic obstructive pulmonary disease; HR, hazard ratio; IQR, interquartile range; NI-PAP, noninvasive positive airway pressure; OME, oral morphine equivalents; OSA, obstructive sleep apnea; risk index, risk stratification index of primary procedure; ZCTA, zip code tabulation area.

^a^ No imputation, and missing data omitted element-wise. Individuals without an OSA screening are given by their reported diagnoses only. Procedure groups, race/ethnicity, and sex categories less than 1% are not reported.

^b^ Cohen *d* for numeric and binary factors and Cohen *w* for categorical factors.

^c^ From *t* tests for numeric and binary factors and χ^2^ tests for categorical factors.

^d^ Top-level clinical classification by organ system; miscellaneous procedures dropped except “other organ transplant.”

^e^ Percentage of adults below the federal poverty line in that individual’s residential area.

^f^ Order for propofol, midazolam, or dexmedetomidine infusion.

^g^ Oral morphine equivalents of intraoperative fentanyl, morphine, hydromorphone, meperidine, and methadone.

A total of 17 682 of all 48 278 CAM-ICU assessments (37%) were positive, and 3637 patients (47%) had delirium at some point in the first 7 days after surgery. Each patient was assessed a median of 4 times (interquartile range, 2-7). Table 2 reports associations of baseline factors with postoperative delirium among patients in the ICU using Cohen *d* or Cohen *w*. The proportion of incident delirium among those with OSA was 44% (897 of 2044) and among those without OSA was 48% (2740 of 5748) (unadjusted risk difference, −0.04; 99% CrI, −0.07 to −0.00). In doubly robust models adjusted for confounders, the protective association of OSA was eliminated and CrIs were narrow enough to exclude a clinically meaningful difference in risk (average treatment effect, −0.01; 99% CrI, −0.04 to 0.03). eTable 6 in the Supplement displays comparison methods and sensitivity analyses. No adjusted analysis generated a point estimate greater than a 0.03 absolute difference in risk or excluded 0 from its CrI or CI. Excluding potential colliders as adjusting variables (eg, age) produced wide CrIs, but otherwise all analyses bounded the increase in risk associated with OSA to less than 5%.

**Table 2.** Association of Baseline Factors With Delirium^a^

Characteristic	Without CAM-ICU (n = 4155)	With CAM-ICU (n = 3637)	Effect size (95% CI)^b^	*P* value^c^
OSA, No. (%)	1147 (28)	897 (25)	0.03 (0.01 to 0.06)	.004
Female sex, No. (%)	1700 (41)	1530 (42)	−0.02 (−0.07 to 0.02)	.30
Age, mean (SD), y	58.2 (15.4)	60.3 (15.0)	−0.14 (−0.18 to 0.09)	<.001
Race/ethnicity, No. (%)				
Unknown	78 (2)	137 (4)	0.08 (0.05 to 0.10)	<.001
Black	563 (14)	592 (16)		
White	3378 (81)	2757 (76)		
Other	136 (3)	151 (4)		
Surgery group, No. (%)^d^				
Other organ transplant	164 (4)	314 (9)	0.11 (0.08 to 0.13)	<.001
Cardiovascular system	1923 (46)	1569 (43)		
Digestive system	686 (17)	651 (18)		
Female genital organs	43 (1)	43 (1)		
Integumentary system	74 (2)	39 (1)		
Musculoskeletal system	581 (14)	473 (13.)		
Nervous system	92 (2)	64 (2)		
Respiratory system	172 (4)	158 (4)		
Urinary system	137 (3)	86 (2)		
Other	283 (7)	240 (7)		
ASA physical status, No. (%)				
1	51 (1)	20 (1)	0.22 (0.19 to 0.24)	<.001
2	700 (17)	296 (8)		
3	1877 (45)	1272 (35)		
4	1491 (36)	1923 (53)		
5	36 (1)	126 (4)		
CAD, No. (%)	1206 (29)	1037 (29)	0.01 (−0.03 to 0.06)	.62
Atrial fibrillation, No. (%)	364 (9)	337 (9)	−0.02 (−0.06 to 0.03)	.44
COPD, No. (%)	550 (13)	557 (15)	−0.06 (−0.10 to 0.02)	.009
CKD, No. (%)	564 (14)	659 (18)	−0.13 (−0.17 to 0.08)	<.001
Dementia, No. (%)	11 (0.3)	32 (1)	−0.08 (−0.13 to 0.04)	<.001
Hypertension, No. (%)	2221 (54)	1748 (48)	0.11 (0.06 to 0.15)	<.001
BMI, mean (SD)	29.3 (7.6)	29.0 (7.5)	0.04 (−0.01 to 0.09)	.09
CCI, median (IQR)	2 (1-4)	3.0 (1-4)	−0.09 (−0.14 to 0.05)	<.001
Risk index, mean (SD), log HR	−0.48 (0.46)	−0.41 (0.49)	−0.14 (−0.18 to 0.09)	<.001
ZCTA poverty, mean (SD), %^e^	16 (10)	17 (10)	−0.05 (−0.11 to 0.00)	.06
Postoperative benzodiazepine order, No. (%)	828 (20)	1327 (37)	−0.38 (−0.42 to 0.33)	<.001
Postoperative sedation, No. (%)^f^	488 (12)	1316 (36)	−0.61 (−0.65 to 0.56)	<.001
Postoperative ventilation, No. (%)	1890 (46)	2904 (80)	−0.75 (−0.80 to 0.71)	<.001
Postoperative NI-PAP, No. (%)	699 (17)	1106 (30)	−0.33 (−0.37 to 0.28)	<.001
Intraoperative OME, mean (SD), mg^g^	96 (60)	88 (63)	0.12 (0.08 to 0.17)	<.001
Midazolam dose, mean (SD), mg	2.5 (2.7)	2.7 (3.2)	−0.09 (−0.14 to 0.05)	<.001

Abbreviations: ASA, American Society of Anesthesiologists; BMI, body mass index (calculated as weight in kilograms divided by height in meters squared); CAD, coronary artery disease; CAM-ICU, Confusion Assessment Method for the intensive care unit; CCI, Charlson Comorbidity Index; CKD, chronic kidney disease; COPD, chronic obstructive pulmonary disease; HR, hazard ratio; IQR, interquartile range; NI-PAP, noninvasive positive airway pressure; OME, oral morphine equivalents; OSA, obstructive sleep apnea; risk index, risk stratification index of primary procedure; ZCTA, zip code tabulation area.

^a^ No imputation, and missing data omitted element-wise. Individuals without an OSA screening are given by their reported diagnoses only. Procedure groups, race/ethnicity, and sex categories less than 1% are not reported.

^b^ Cohen *d* for numeric and binary factors and Cohen *w* for categorical factors (identical to Cramer V for 2 groups).

^c^ From *t* tests for numeric and binary factors and χ^2^ tests for categorical factors.

^d^ Top-level clinical classification by organ system; miscellaneous procedures dropped except “other organ transplant.”

^e^ Percentage of adults below the federal poverty line in that individual’s residential area.

^f^ Order for propofol, midazolam, or dexmedetomidine infusion.

^g^ Oral morphine equivalents of intraoperative fentanyl, morphine, hydromorphone, meperidine, and methadone.

**Figure 2.**
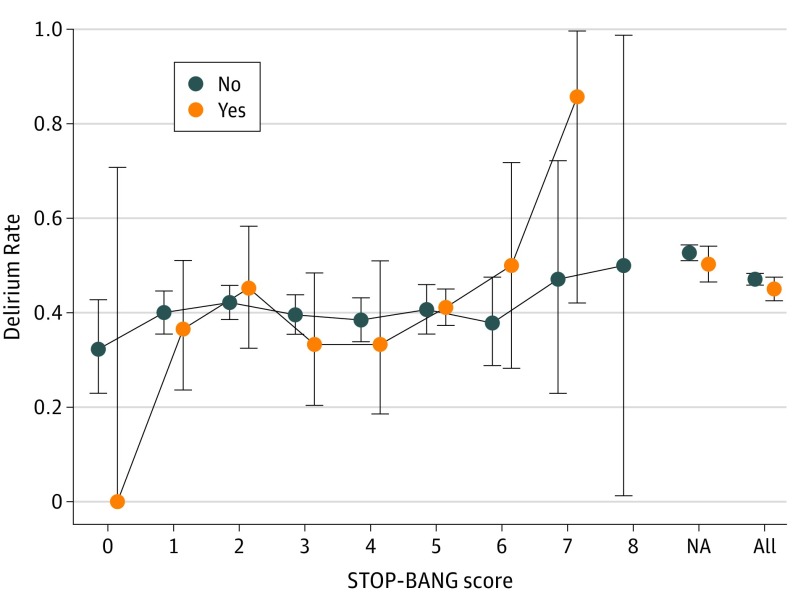
Delirium Rate by STOP-BANG (Snoring, Tiredness, Observed Apnea, Blood Pressure, Body Mass Index, Age, Neck Circumference and Gender) Score and Obstructive Sleep Apnea Diagnosis Delirium incidence proportion stratified by STOP-BANG score and preexisting obstructive sleep apnea diagnosis (no vs yes). Vertical bars are pointwise 95% Clopper-Pearson CIs. Within no diagnosis, unadjusted odds ratio per point of STOP-BANG, 1.01. NA indicates not applicable.

We also assessed for an association between postoperative delirium and risk of undiagnosed OSA via quantitative STOP-BANG score. The incidence proportion of delirium stratified by OSA diagnosis and STOP-BANG score is plotted in Figure 2. After adjustment, STOP-BANG was not associated with delirium among the subset of patients without an OSA diagnosis (log odds ratio, −0.01; 99% CI, −0.08 to 0.11; population effect of setting STOP-BANG to 0, 0.00; 99% CrI, −0.04 to 0.06).

Evaluation of the propensity models showed that the variables most associated with OSA were weight, sex, hypertension, surgery performed, assessment location, and surgery after routine STOP-BANG implementation in 2013. The full set of variable importance metrics and logistic regression coefficients is presented in eTable 7 and eTable 8 in the Supplement. The overall C statistic for predicting OSA was 0.835 with minimal optimism (out of sample C, 0.835; 99% CrI, 0.807-0.862), suggesting little overfitting. Propensity score balance diagnostics are presented in eAppendix 8 in the Supplement; standardized mean differences were low for all variables (<10^−5^). Overlap between the OSA and non-OSA groups was good, with 74% (1521 of 2044) of exposed samples in a broadly overlapping region (eFigure 5 in the Supplement). The out-of-sample C statistic predicting delirium was 0.713 (99% CI, 0.679-0.745), suggesting a moderate-quality fit.

In the analysis of the outcome of adherence to PAP therapy, the proportion of delirium among those with routine adherence was 44% (227 of 511) and among those with nonadherence was 44% (150 of 338), with an unadjusted 99% CI on the average treatment effect of −0.09 to 0.09. Adjustment made minimal difference (average treatment effect, −0.00; 99% CrI, −0.07 to 0.07). The variables most associated with adherence to PAP therapy were weight and race/ethnicity. The in-sample C statistic was 0.771 and the cross-validated C statistic was 0.644 (99% CI, 0.540-0.738), suggesting a weak overall fit and modest overfitting.

## Discussion

In this large, retrospective surgical cohort, we found that postoperative delirium in the ICU was slightly less prevalent among patients with OSA. After adjustment for measured confounding factors, there was no longer a significant association. Our data strongly contrast with prior work and quantitatively exclude our hypothesis that OSA increases the risk for postoperative delirium by a meaningful amount (<5% absolute difference with a background rate of 47%). If these results are replicated, interventional studies targeting adherence to PAP therapy are therefore unlikely to substantially prevent delirium.

We offer several explanations for this finding. First, OSA could simply be a less important risk factor for postoperative delirium than previously believed. The literature draws a somewhat tenuous connection between OSA and postoperative adverse outcomes, with some studies finding (unadjusted) negative associations.^29^ An association of treatment with PAP therapy and cardiovascular outcomes is supported by low-quality evidence.^16,17^ A recent prospective study with sleep studies in all participants found an association between severe undiagnosed OSA and a composite of postoperative cardiovascular outcomes.^11^ It also found a nonsignificant but clinically meaningful point estimate association with postoperative delirium. Delirium was infrequent in that sample, and precision was too low to exclude moderate effect sizes. In addition, the association between the effects of severe unrecognized OSA and diagnosed OSA is not a priori clear. Thresholds of OSA severity may be necessary to elucidate strong associations.^30^ We also observed no association between STOP-BANG scores (which are associated with OSA severity^11^) and postoperative delirium among those without an OSA diagnosis but with wide uncertainty. The clinical context may have evolved over time; our result of no association is in the presence of warning bands that say “high-risk OSA” and automatically prompted admission order sets related to OSA. Although there is little evidence that any specific treatments in response to these warnings prevent delirium, it is possible that these labels led to avoidance of risk-increasing events of hypoxia and sedating drug use.

### Strengths and Limitations

This study has some strengths, including the relatively large sample size, the high rate of participation in structured preoperative evaluation, and the use of structured CAM-ICU evaluations rather than determining delirium diagnoses from administrative data. Our delirium assessments occurred only in the ICU, so incident delirium after transfer to the wards would have been missed, but this is likely a small fraction of postoperative delirium in this population. Although we failed to capture some potential covariates discussed above and have no measure of OSA severity, our analyses take advantage of the large sample to flexibly include many covariates. We analyzed the data with several methods and obtained similar results, and the methods used were nonparametric, reducing the dependence on statistical assumptions and model specifications.

This study has some limitations. We targeted our analysis toward causal quantities (treatment effects), as this facilitates understanding the magnitude of associations and biases as well as the clinical importance of the problem. However, our study is a typical retrospective cohort. Other than adjustment for many confounders and the likely higher quality of anesthesia clinic assessments vs other sources of clinical history, there is no natural experiment to give a strong causal implication.

Our single-center results can only be cautiously generalized. We include diverse surgical procedures, and the mix of these procedures will be different elsewhere; we do not advocate the fitted models for assessing the risk of postoperative delirium in other contexts. Patients undergoing neurosurgery, those with long preoperative ICU stays, and those in the medical ICU after surgery were excluded. Delirium rates in our study are 10-fold higher than in the report by Chan and colleagues.^11^ Other institutions discovered highly variable rates of ICU delirium.^31^ One national database found similar rates and accuracy at predicting ICU delirium, although the populations and included factors are not comparable.^32^ Only 13% of patients in the ICU in our study were never assessed by CAM-ICU, and our sensitivity analyses suggest that missing CAM-ICU assessments do not play a major role in our findings. As explored in eAppendix 3 and eFigure 2 in the Supplement, individuals who were not assessed with the CAM-ICU were likely a mixture of relatively well patients with short ICU stays and very ill patients who died without being assessable. The median of 4 assessments per patient suggests that the CAM-ICU was routinely performed. Our high delirium rates could reflect more consistent CAM-ICU performance, greater sensitivity at detecting less severe delirium, or surgery performed in a population that is much more prone to delirium, limiting generalizability.

In addition, several sources of bias owing to incomplete data and the observational nature of our study could also explain our null findings. Differential OSA measurement error could induce a protective bias that matches the information in Table 1 and Table 2. It is plausible that individuals evaluated with less accuracy were not as carefully evaluated for OSA and that these individuals had higher rates of delirium; for example, patients with altered mental status or who underwent urgent surgical procedures were likely not meaningfully screened. This likely explains the much higher nonscreening rate in the ICU population (26%) than in the overall population (8%). However, even in the elective preoperative clinic group, no association was observed after adjustment. We also did not find an association when splitting the cohort into prescreening and postscreening era sets or when ignoring STOP-BANG screening, mitigating likely bias magnitude. We would expect diagnosed OSA to be specific (and include more severe cases) if not sensitive, which could inflate the effect size. We also did not observe a dose-response association with STOP-BANG values (and therefore fraction of OSA) in the undiagnosed population. Although exposure misclassification tends to bias toward the null, the direction of effect can be unpredictable, especially with misclassified confounders.^33^ Although the rate of patients receiving the CAM-ICU was high (87%), mismeasurement or selective reporting of delirium could also bias our results.

Selection bias in surgical procedures and ICU admission associated with OSA (less ill patients with OSA admitted to the ICU or offered less invasive procedures) may cancel out an opposite signed direct association of OSA. However, the risk stratification index differed only minimally between patients with and without OSA who were admitted to the ICU. Although we used a large set of adjustment variables, we do not have accurate measures of individual socioeconomic variables (such as educational level) that are likely associated with both OSA diagnosis and delirium; we have only residence location proxies. In addition to selection bias, the adjusted models have potential collider bias. Because the ailments being treated by surgery may be caused indirectly by OSA (eg, hypertension, atrial fibrillation, and cardiac disease), the adjusted estimate could be falsely reassuring, paralleling the protective association of obesity “adjusted for” cardiovascular disease.^34,35^ However, collider biases from similar causal models tend to be smaller than confounding biases,^36,37^ and sensitivity analyses excluding the colliders produced similar results.

As alluded to above, we have very limited measurement of postoperative therapies, and these may have created the null association we observe. That is, one could posit that a direct negative association of OSA is counterbalanced by beneficial associations of treatments downstream of OSA; dissecting these mediating associations is very difficult (especially of a null association). Our data set does not include supplemental oxygen use or postoperative medication administration to confirm this hypothesis, although our sensitivity analysis with benzodiazepine and opioid use did not find an association. Our data can address the risk of delirium only in the context of the care provided, which is the relevant consideration for patients and researchers. In addition, the total association of OSA is the clinically most important quantity. If the presence of OSA reduces the use of medications that induce delirium, that is a real effect.

Despite the large size of our cohort, the sample size for estimating the association of delirium and preoperative adherence to PAP therapy was small, and the estimates had broad CrIs including substantial risk increases and decreases. Similar cautions about bias as given above for OSA apply to the association of PAP therapy with delirium. Many patients did not have a recorded response to the question about PAP therapy use, creating a stronger risk of bias due to missing data. Confounding by indication may falsely diminish the protective association of PAP therapy; patients with the most severe symptoms and largest symptom benefit are those most likely to have PAP therapy prescribed and are those most likely to adhere to it.^38,39^

## Conclusions

This retrospective cohort of patients admitted to the ICU after surgery found a decreased rate of delirium among patients with OSA, which was eliminated by adjustment for confounding factors. Validity threats from measurement errors, unmeasured confounding, or differences in postoperative care could mask a true positive association, but a large increase in risk is unlikely. We found a minimal association with preoperative adherence to PAP therapy, with large uncertainty. Our work suggests that additional high-quality data linking these outcomes are needed before interventional trials of PAP therapy and delirium.

## Notes

Supplement. **eAppendix 1.** Description of Anesthesia Assessment and Coding of Predictor Variables **eAppendix 2.** Imputation Procedure **eTable 1.** Baseline Factors by OSA Status in Non-ICU and Overall Surgical Population **eFigure 1.** Overall Analytic Flow **eAppendix 3.** Exploration of ICU Admission and Missing Delirium Status as Sources of Bias **eTable 2.** Assessment Rate by ICU After Filtering to Date After 11/1/2012 **eFigure 2.** Data Flow Before Applying ICU and Time Filters **eFigure 3.** Histogram of Propensity to ICU Admission in Admitted and Unadmitted Patients **eFigure 4.** Histogram of Propensity to CAM-ICU Assessment Among ICU Patients in Assessed and Unassessed Patients **eTable 3.** Covariates by CAM-ICU Assessment Status and OSA Status **eAppendix 4.** Propensity Score Generation and Primary Analysis **eFigure 5.** Histogram of OSA Propensity Score by OSA Status in the Entire Surgical Population **eAppendix 5.** Sensitivity Analyses and Alternative Analytic Approaches **eAppendix 6.** Hyperparameters, Tuning, and Variable Importance **eAppendix 7.** STOP-BANG Contrasts **eTable 4.** Covariance and Marginal Distribution of STOP-BANG Elements **eTable 5.** Prevalence of OSA Diagnosis and Rate of Missing STOP-BANG Criteria by Surgical Category **eTable 6.** Comparison of Effect Estimates From Sensitivity Analyses **eTable 7.** Logistic Regression Coefficients **eTable 8.** Variable Importance Metrics for Propensity Models **eAppendix 8.** Propensity Score Balance Diagnostics **eReferences**
