# Supplementary material for: Association Between Preoperative Obstructive Sleep Apnea and Preoperative Positive Airway Pressure With Postoperative Intensive Care Unit Delirium
Source: JAMA Netw Open. 2020 Apr 20;3(4):e203125. doi: 10.1001/jamanetworkopen.2020.3125 (PMC7171553; doi:10.1001/jamanetworkopen.2020.3125)
Supplement: Supplement. — eAppendix 1. Description of Anesthesia Assessment and Coding of Predictor Variables eAppendix 2. Imputation Procedure eTable 1. Baseline Factors by OSA Status in Non-ICU and Overall Surgical Population eFigure 1. Overall Analytic Flow eAppendix 3. Exploration of ICU Admission and Missing Delirium Status as Sources of Bias eTable 2. Assessment Rate by ICU After Filtering to Date After 11/1/2012 eFigure 2. Data Flow Before Applying ICU and Time Filters eFigure 3. Histogram of Propensity to ICU Admission in Admitted and Unadmitted Patients eFigure 4. Histogram of Propensity to CAM-ICU Assessment Among ICU Patients in Assessed and Unassessed Patients eTable 3. Covariates by CAM-ICU Assessment Status and OSA Status eAppendix 4. Propensity Score Generation and Primary Analysis eFigure 5. Histogram of OSA Propensity Score by OSA Status in the Entire Surgical Population eAppendix 5. Sensitivity Analyses and Alternative Analytic Approaches eAppendix 6. Hyperparameters, Tuning, and Variable Importance eAppendix 7. STOP-BANG Contrasts eTable 4. Covariance and Marginal Distribution of STOP-BANG Elements eTable 5. Prevalence of OSA Diagnosis and Rate of Missing STOP-BANG Criteria by Surgical Category eTable 6. Comparison of Effect Estimates From Sensitivity Analyses eTable 7. Logistic Regression Coefficients eTable 8. Variable Importance Metrics for Propensity Models eAppendix 8. Propensity Score Balance Diagnostics eReferences [file jamanetwopen-3-e203125-s001.pdf]

Supplementary Online Content

King CR, Fritz BA, Escallier K, et al. Association between preoperative obstructive sleep apnea and preoperative positive airway pressure with postoperative intensive care unit delirium. *JAMA Netw Open.* 2020;3(4):e203125.
doi:10.1001/jamanetworkopen.2020.3125

eAppendix 1. Description of Anesthesia Assessment and Coding of Predictor Variables
eAppendix 2. Imputation Procedure
eTable 1. Baseline Factors by OSA Status in Non-ICU and Overall Surgical Population
eFigure 1. Overall Analytic Flow
eAppendix 3. Exploration of ICU Admission and Missing Delirium Status as Sources of Bias
eTable 2. Assessment Rate by ICU After Filtering to Date After 11/1/2012
eFigure 2. Data Flow Before Applying ICU and Time Filters
eFigure 3. Histogram of Propensity to ICU Admission in Admitted and Unadmitted Patients
eFigure 4. Histogram of Propensity to CAM-ICU Assessment Among ICU Patients in Assessed and Unassessed Patients
eTable 3. Covariates by CAM-ICU Assessment Status and OSA Status
eAppendix 4. Propensity Score Generation and Primary Analysis
eFigure 5. Histogram of OSA Propensity Score by OSA Status in the Entire Surgical Population
eAppendix 5. Sensitivity Analyses and Alternative Analytic Approaches
eAppendix 6. Hyperparameters, Tuning, and Variable Importance
eAppendix 7. STOP-BANG Contrasts
eTable 4. Covariance and Marginal Distribution of STOP-BANG Elements
eTable 5. Prevalence of OSA Diagnosis and Rate of Missing STOP-BANG Criteria by Surgical Category
eTable 6. Comparison of Effect Estimates From Sensitivity Analyses
eTable 7. Logistic Regression Coefficients
eTable 8. Variable Importance Metrics for Propensity Models
eAppendix 8. Propensity Score Balance Diagnostics
eReferences

This supplementary material has been provided by the authors to give readers additional information about their work.

© 2020 King CR et al. *JAMA Network Open.*

eAppendix 1. Description of Anesthesia Assessment and Coding of Predictor Variables

The anesthesia assessment was performed by a nurse or nurse practitioner trained in preoperative evaluation or by an anesthesiology resident. After history, physical exam, and laboratory records were collated, these evaluations were reviewed with an attending anesthesiologist. Unless noted otherwise values used below were selected from a drop-down-menu or check-box by the anesthesia provider or nurse in the Metavision system.

The authors had complete access to the anesthesia information system, Metavision. The complete set of anesthesia cases entered into metavision during the study timeframe was extracted. These contained case ID's, dates of service, a medical record number, and a master record number. The set of medical record numbers was submitted to extract all matching administrative records including demographics, hospitalization dates, billing codes, and ICU admission and discharge times. Preoperative assessment and hospitalization encounter identifiers were submitted to obtain nursing flowsheets. Laboratory values were queried on the same set of patients in a 6 month interval surrounding the date of service. All metavision records were linked to a master record number; approximately 0.5% contained invalid medical record numbers and were matched by DoS to the hospitalization.

Pre-anesthesia evaluations from Metavision were linked for all cases from 6 months prior to the index case to 1 week after. Numerous evaluations were incomplete. Evaluations were marked as invalid if 1) completed on DoS with no findings and no ASA-PS 2) no findings but ASA-PS >2 3) CCI derived from administrative data present-on-admission diagnoses >2 higher than implied by evaluation 4) No location for evaluation. The presence of a chronic condition was marked as positive (or the worst-level for ordinal conditions) if any evaluations contained it as positive. Evaluation characteristics were assigned based on the evaluation with the least discrepancy by euclidean distance from the overall set of chronic conditions. STOP questionnaire and Neck measurements were entered separately in the EHR nursing flowsheets, and the most recent preoperative values were taken. For patients with zero completed preoperative evaluations, chronic conditions were taken from postoperative evaluations if present. This was relatively common in emergent cases, but very rare in elective ones used in the analysis. Vital signs were taken from the most recent non-missing values. OSA diagnosis was also obtained from ICD diagnosis codes associated with the admission, using ICD9CM codes 327.2, 780.51, 780.53, 780.57 and ICD10 codes G47.3 and R06.81. Nursing flowsheets for the CAM-ICU were categorized as "Positive" or "Negative" (no free text).

The following variables were included as predictors in propensity models. Variables marked as ordinal are treated as continuous by BART (tree-based methods functionally treat all numeric and ordinal variables the same), but used as categorical predictors in logistic regression and imputed as ordinal outcomes in MICE. A first pass of MICE was used to define conditional expected values, and quantitative variables > 5 standard deviations from their expected values were marked as missing. Heights > 213 cm or <120cm, weights < 20 kg or > 250 kg, systolic blood pressure < 25 mmHg or > 300 mmHg, diastolic blood pressures <10 mmHg, > 160 mmHg, neck circumferences < 20 cm or > 60 cm were marked as missing (data entry error).

Surgery type (Surg_Type), categorical. Age, continuous. Sex, categorical. Race, categorical. Height, continuous. Weight, continuous. Charlson comorbidity index (CCI), continuous. Functional capacity, 5 level ordinal. ASA physical status (ASA), ordinal. Preoperative pain, continuous. Hypertension diagnosis (HTN), binary. Coronary artery disease diagnosis (CAD), binary. Prior myocardial infarction (CAD_PRIORMI), binary. Congestive heart failure diagnosis (CHF), binary. Diastolic dysfunction (CHF_Diastolic_Function), 4 level ordinal. Left ventricular ejection fraction (LVEF), binary indicator for unmeasured plus continuous (qualitative categories “normal” “mildly reduced” “moderately reduced” and severely reduced” were mapped to 65%, 55%, 40%, and 25% respectively). Valvular disease, ordinal 5 levels. Valve affected, 4 binary variables. Atrial fibrillation (AFIB), ordinal 4 levels (isolated, paroxysmal, persistent, permanent). Pacemaker or ICD implanted (PPM_ICD), binary. Diagnosis of TIA or stroke (CV_TIA_STROKE), binary. Diagnosis of peripheral arterial disease (PAD), binary. History of deep vein thrombosis (DVT) or pulmonary embolism (PE), binary. Diabetes, non-insulin dependent, binary. Insulin use,

© 2020 King CR et al. *JAMA Network Open.*

categorical (none, previous, current, pump). Diagnosis of CKD, binary. Dialysis use, categorical (never, previously only, current PD, current HD). Diagnosis of pulmonary hypertension (PHTN), binary. Diagnosis of COPD, binary. Diagnosis of asthma, binary. Diagnosis of cirrhosis, binary, Diagnosis of cancer excluding isolated skin cancer, binary. Diagnosis of GERD, binary. Diagnosis of dementia. Binary. History of coombs-positive blood reaction, binary. History of smoking, binary. Preoperative systolic blood pressure, continuous. Preoperative diastolic blood pressure, continuous. Year of service, continuous. Assessed before STOP-BANG screening (before_screening), binary. ICU admission (is_ICU), binary. AHRQ CCS category - categorical.

The following local average quantitative variables were extracted based on the reported ZCTA and census (2010) or american community survey (2016) data: total population, fraction urban, fraction black, white, asian, and hispanic, fraction of housing vacant, fraction of adults employed, fraction of adults living in poverty, fraction of adults with education of less than high school, high school, and college. To reduce colinearity the fraction college and fraction white were excluded (they are functionally reference categories).

Variables computed from the above (ideal body weight, BMI) were excluded during imputation to reduce issues with colinearity and passively imputed (i.e. recalculated).

Procedure codes were retrieved for 96% of ICU patients. Addresses were linked to a ZTCA for 67% of patients, with the unlinked addresses primarily PO boxes and business addresses.

Intraoperative and recovery area (for patients going to PACU before ICU) opioid doses were standardized to oral morphine equivalents as follows: oxycodone * 1.5, IV meperidine *0.3, IV morphine *3, IV hydromorphone *11.5, IV fentanyl (mcg) *0.15, IV methadone * 4.5. Sufentanil and remifentanil were discarded because of their short duration of action. No other opioids were encountered intraoperatively. The only intraoperative benzodiazepine used was midazolam. Postoperative sedation was defined as infusions of midazolam, propofol, or dexmedetomidine. Postoperative medication Postoperative intubation was extracted by 1) intubation or in-situ airway intraoperatively without an extubation mark and 2) ICU ventilator flowsheet events. Postoperative non-invasive positive pressure ventilation was detected by ventilation settings of "cpap," "bipap," "avaps," "vpap," and spelling variants not prior to an extubation event. We were unable to link postoperative medication *administrations* and instead rely on postoperative medication *orders* since benzodiazepines are not frequently ordered as as-needed medications at our institution without a belief that they will be necessary.

eAppendix 2. Imputation Procedure

Procedure codes and CCS were not imputed; “missing procedure codes” was created as an additional factor level and risk stratification index was imputed as a usual numerical variable. CCS categories and surgical service categories with fewer than 40 occurrences in the dataset after all filters were collapsed to an “other” category. Missing covariate data was multiple imputed using chained regression [1] with 30 replicates. Imputation included OSA and delirium as predictor covariables. A multiple-impute-then-delete strategy [2,3] was used for all analyses (i.e. imputed outcomes were not used). OSA and PAP use were included in the procedure, but similarly never imputed. In sensitivity analyses we experimented with imputation using only the analytic sample, the entire surgical population, and excluding postoperative data.

© 2020 King CR et al. *JAMA Network Open.*

eTable 1. Baseline Factors by OSA Status in Non-ICU and Overall Surgical Population
See Table 1 for definitions.

	ICU -				Overall			
	OSA -	OSA +	effect size (95% CI)	p	OSA -	OSA +	effect size (95% CI)	p
	n=53167	n=15767			n=58915	n=17811		
Female Sex			0.29 (0.27 0.31)	< 1e-05			0.29 (0.27 0.30)	< 1e-05
Yes	29381 (55.3%)	6450 (40.9%)			31941 (54.2%)	7120 (40.0%)		
surgery type			0.070 (0.070 0.080)	< 1e-05			0.070 (0.060 0.080)	< 1e-05
Operations on the cardiovascular system	3286 (6.2%)	1039 (6.6%)			5808 (9.9%)	2009 (11.3%)		
Operations on the digestive system	7666 (14.4%)	2049 (13.0%)			8692 (14.8%)	2360 (13.3%)		
Operations on the female genital organs	5343 (10.0%)	962 (6.1%)			5408 (9.2%)	983 (5.5%)		

© 2020 King CR et al. *JAMA Network Open.*

Operations on the hemic and lymphatic system	832 (1.6%)	276 (1.8%)			853 (1.4%)	286 (1.6%)		
Operations on the integumentary system	1685 (3.2%)	374 (2.4%)			1766 (3.0%)	406 (2.3%)		
Operations on the male genital organs	688 (1.3%)	312 (2.0%)			703 (1.2%)	321 (1.8%)		
Operations on the musculoskeletal system	12503 (23.5%)	3988 (25.3%)			13242 (22.5%)	4303 (24.2%)		
Operations on the nervous system	3871 (7.3%)	1411 (8.9%)			3994 (6.8%)	1444 (8.1%)		
Operations on the nose; mouth; and pharynx	1403 (2.6%)	418 (2.7%)			1436 (2.4%)	431 (2.4%)		

© 2020 King CR et al. *JAMA Network Open.*

Operations on the respiratory system	2762 (5.2%)	765 (4.9%)			3017 (5.1%)	840 (4.7%)		
Operations on the urinary system	4314 (8.1%)	1389 (8.8%)			4479 (7.6%)	1447 (8.1%)		
Other	8814 (16.6%)	2784 (17.7%)			9517 (16.2%)	2981 (16.7%)		
ASA			0.21 (0.21 0.22)	< 1e-05			0.21 (0.20 0.21)	< 1e-05
1	4551 (8.6%)	183 (1.2%)			4618 (7.8%)	187 (1.0%)		
2	26621 (50.1%)	5403 (34.3%)			27435 (46.6%)	5585 (31.4%)		
3	18647 (35.1%)	9026 (57.2%)			20876 (35.4%)	9946 (55.8%)		
4	3146 (5.9%)	1120 (7.1%)			5638 (9.6%)	2042 (11.5%)		
5	202 (0.4%)	35 (0.2%)			348 (0.6%)	51 (0.3%)		
Race			0.080 (0.070 0.090)	< 1e-05			0.080 (0.070 0.090)	< 1e-05
Unknown	596 (1.1%)	110 (0.7%)			770 (1.3%)	151 (0.8%)		

© 2020 King CR et al. *JAMA Network Open.*

Black	10851 (20.4%)	2424 (15.4%)			11756 (20.0%)	2674 (15.0%)		
White	39398 (74.1%)	12912 (81.9%)			43819 (74.4%)	14626 (82.1%)		
Other	2322 (4.4%)	321 (2.0%)			2570 (4.4%)	360 (2.0%)		
CAD								
Yes	4281 (8.1%)	2905 (18.4%)	-0.34 (-0.36 - 0.33)	< 1e-05	5737 (9.7%)	3692 (20.7%)	-0.34 (-0.36 - 0.32)	< 1e-05
Atrial fib								
Yes	1407 (2.6%)	1214 (7.7%)	-0.27 (-0.28 - 0.25)	< 1e-05	1824 (3.1%)	1498 (8.4%)	-0.26 (-0.28 - 0.25)	< 1e-05
COPD								
Yes	3197 (6.0%)	1849 (11.7%)	-0.22 (-0.24 - 0.20)	< 1e-05	3882 (6.6%)	2271 (12.8%)	-0.23 (-0.24 - 0.21)	< 1e-05
CKD								
Yes	4127 (7.8%)	2491 (15.8%)	-0.27 (-0.29 - 0.26)	< 1e-05	4877 (8.3%)	2964 (16.6%)	-0.28 (-0.29 - 0.26)	< 1e-05
Dementia								

© 2020 King CR et al. *JAMA Network Open.*

Yes	271 (0.5%)	110 (0.7%)	-0.030 (-0.040 -0.010)	0.01	301 (0.5%)	123 (0.7%)	-0.020 (-0.040 -0.010)	0.0089
age (years)								
Mean (SD)	51 (17)	59 (13)	-0.48 (-0.50 - 0.46)	< 1e-05	52 (17)	59 (13)	-0.46 (-0.47 - 0.44)	< 1e-05
Median [Q1, Q3]	53 [38, 64]	60 [52, 68]			54 [39, 65]	61 [52, 68]		
BMI (mk/kg**2)								
Mean (SD)	28 (6.8)	34 (8.7)	-0.83 (-0.85 - 0.81)	< 1e-05	28 (6.8)	34 (8.6)	-0.83 (-0.85 - 0.81)	< 1e-05
Median [Q1, Q3]	27 [23, 32]	33 [28, 39]			27 [23, 31]	33 [28, 39]		
Missing	1530 (2.9%)	101 (0.6%)			2141 (3.6%)	156 (0.9%)		
HTN								
Yes	18349 (34.5%)	10968 (69.6%)	-0.74 (-0.76 - 0.72)	< 1e-05	20863 (35.4%)	12423 (69.7%)	-0.72 (-0.74 - 0.71)	< 1e-05
CCI								

© 2020 King CR et al. *JAMA Network Open.*

Mean (SD)	2.0 (2.2)	2.8 (2.2)	-0.35 (-0.37 - 0.33)	< 1e-05	2.1 (2.2)	2.9 (2.2)	-0.34 (-0.36 - 0.32)	< 1e-05
Median [Q1, Q3]	1.0 [0.0, 3.0]	2.0 [1.0, 4.0]			2.0 [0.0, 3.0]	2.0 [1.0, 4.0]		
Missing	550 (1.0%)	183 (1.2%)			611 (1.0%)	201 (1.1%)		
Risk strat index 1								
Mean (SD)	-0.64 (0.58)	-0.66 (0.61)	0.030 (0.010 0.050)	0.00081	-0.62 (0.57)	-0.63 (0.60)	0.030 (0.010 0.050)	0.0025
Median [Q1, Q3]	-0.66 [-1.0, -0.25]	-0.75 [-1.0, -0.25]			-0.64 [-1.0, -0.25]	-0.66 [-0.98, -0.25]		
Missing	5565 (10.5%)	1931 (12.2%)			5776 (9.8%)	2011 (11.3%)		
ZTCA poverty								
Mean (SD)	16 (10)	15 (9.4)	0.12 (0.10 0.14)	< 1e-05	16 (10)	15 (9.4)	0.11 (0.090 0.13)	< 1e-05
Median [Q1, Q3]	14 [8.2, 22]	13 [7.8, 20]			14 [8.4, 22]	14 [8.0, 20]		
Missing	16284 (30.6%)	4906 (31.1%)			18149 (30.8%)	5558 (31.2%)		

© 2020 King CR et al. *JAMA Network Open.*

ZTCA employment								
Mean (SD)	0.92 (0.051)	0.92 (0.045)	-0.11 (-0.13 - 0.080)	< 1e-05	0.92 (0.051)	0.92 (0.045)	-0.10 (-0.12 - 0.080)	< 1e-05
Median [Q1, Q3]	0.93 [0.90, 0.95]	0.93 [0.91, 0.95]			0.93 [0.90, 0.95]	0.93 [0.91, 0.95]		
Missing	16284 (30.6%)	4906 (31.1%)			18149 (30.8%)	5558 (31.2%)		
ZTCA housing								
Mean (SD)	11 (7.1)	10 (6.4)	0.12 (0.10 0.14)	< 1e-05	11 (7.1)	10 (6.5)	0.11 (0.090 0.13)	< 1e-05
Median [Q1, Q3]	9.2 [6.8, 13]	9.0 [6.5, 12]			9.2 [6.8, 13]	9.1 [6.6, 12]		
Missing	16284 (30.6%)	4906 (31.1%)			18149 (30.8%)	5558 (31.2%)		
Postop benzodiazepine								
Yes	1880 (3.5%)	457 (2.9%)	0.040 (0.020 0.050)	4.3e-05	3491 (5.9%)	1001 (5.6%)	0.010 (0.0 0.030)	0.12
Postop sedation								

© 2020 King CR et al. *JAMA Network Open.*

Yes	717 (1.3%)	182 (1.2%)	0.020 (0.0 0.030)	0.049	2066 (3.5%)	637 (3.6%)	0.0 (- 0.020 0.010)	0.66
Postop intubation								
Yes	2458 (4.6%)	617 (3.9%)	0.030 (0.020 0.050)	7.5e-05	5932 (10.1%)	1937 (10.9%)	-0.030 (-0.040 -0.010)	0.0023
No	50709 (95.4%)	15150 (96.1%)			52983 (89.9%)	15874 (89.1%)		
Postop NIPPV								
Yes	840 (1.6%)	380 (2.4%)	-0.060 (-0.080 -0.050)	< 1e-05	1895 (3.2%)	1130 (6.3%)	-0.16 (- 0.18 - 0.14)	< 1e-05
OME								
Mean (SD)	56 (40)	58 (40)	-0.040 (-0.060 -0.020)	1.2e-05	60 (43)	63 (45)	-0.070 (-0.080 -0.050)	< 1e-05
Median [Q1, Q3]	50 [30, 74]	52 [30, 75]			53 [31, 77]	53 [33, 81]		
Pre and intraop midazolam dose								
Mean (SD)	2.2 (1.9)	2.1 (2.5)	0.060 (0.040 0.080)	< 1e-05	2.3 (2.0)	2.1 (2.5)	0.050 (0.040 0.070)	< 1e-05

© 2020 King CR et al. *JAMA Network Open.*

Median [Q1, Q3]	2.0 [1.0, 3.0]	2.0 [1.0, 3.0]			2.0 [1.0, 3.0]	2.0 [0.0, 3.0]		

© 2020 King CR et al. *JAMA Network Open.*

eFigure 1. Overall Analytic Flow

See eAppendix 4 for primary analysis plan. After data filters (age, general anesthesia, anesthesia assessment completed, icu admission) are applied multiple imputation by chained regression is applied to generate 30 "completed" datasets. Within each round a propensity score is calculated. The data are then re-filtered to remove patients whose outcomes were unknown. An effect size estimate for OSA is calculated within each completed dataset by BART, BCF, matching, regression. For each method, the results are then combined across the 30 imputations by Rubin's rules for matching and regression and by mixing the posterior simulations for BART and BCF. Confidence intervals generated by Wald method for regression and matching, and highest-posterior-density credible intervals are created for BART and BCF. Ovals in figure are a vertical ellipsis. Sensitivity analyses contain variations of this flow.

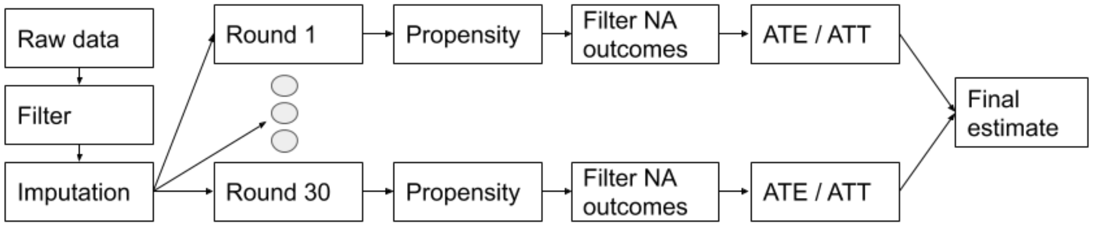

© 2020 King CR et al. *JAMA Network Open.*

eAppendix 3. Exploration of ICU Admission and Missing Delirium Status as Sources of Bias

We found that among all patients admitted to the ICU postoperatively, there was a substantial fraction of never-assessed patients (zero documented CAM-ICU). To reduce concerns from bias caused by missing data, we investigated the patterns of missing assessments. First, we found that although CAM-ICUs were performed during the specified time period (8/2012-8/2106), they were not routinely documented as discrete data until 11/2012 (18% pre and 70% post). We had initially planned to include data through 2018; however, the informatics infrastructure unexpectedly changed after our initial data pull in 2016 and removed the ability to feasibly query data. We therefore excluded cases before that time. Second, we found that the rate of performing CAM-ICU varied dramatically by ICU. Patients in the neurosurgical ICU had CAM-ICUs performed in a low fraction of cases (1.8% with year to year variation); other ICUs had less extreme fractions (cardiac care 66%, bone marrow transplant 55%). The remaining ICUs (cardiothoracic surgery, surgical-burn, medical) had high factions of assessment (84-90%). We also found that many patients in the non-surgical ICUs had relatively minor surgical procedures (tracheostomy, gastric tube placement, wound re-debridement, dressing change under anesthesia). The attribution of delirium in patients with substantial prior ICU stays as "postoperative" would be ambiguous; however, we did not wish to exclude all patients with prior ICU stays as it was not uncommon to have brief pre-admissions to the ICU before major surgery. We therefore excluded patients with >= 6 days of prior ICU stay, as this would eliminate the majority of these minor procedures in severely ill patients. Patients with an *index* surgery without a prior ICU stay remain in the dataset even if they re-visit the OR in the subsequent 7 days. A version of Figure 1 *without* these steps is provided in eFigure 2. These filters are a deviation from the analysis plan but primarily refine the included population rather than change the analysis. That is, because almost all excluded patients would have been excluded later (by virtue of lacking a delirium assessment) the actual analytic population is little changed, but the results can be stated with more confidence that selection bias in missing assessments did not play a major role.

© 2020 King CR et al. *JAMA Network Open.*

eTable 2. Assessment Rate by ICU After Filtering to Date After 11/1/2012

icu_unit	assessment rate	n total
10400 ICU	0.0181	2535
4400 ICU	0.904	5038
5600 ICU	0.849	3952
8200 ICU	0.665	242
83 CTICU	0.904	477
8300 ICU	0.744	129
8400 ICU	0.878	74
8900 ICU	0.52	25
8900 Oncology ICU	0.4	5

© 2020 King CR et al. *JAMA Network Open.*

eFigure 2. Data Flow Before Applying ICU and Time Filters
(pre-specified plan), compare to Figure 1.

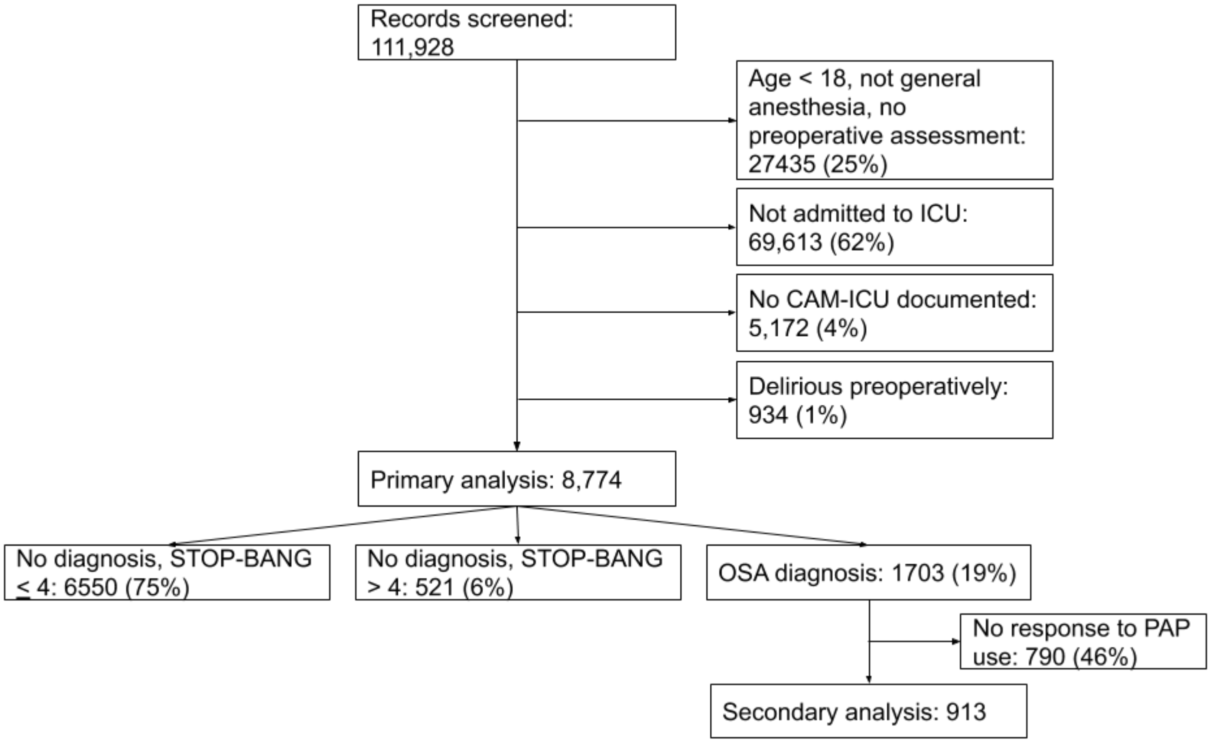

After applying these restrictions, to explore the possibility of OSA changing ICU admission rates or CAM-ICU performance, we then undertook a propensity analysis of CAM-ICU documentation in 2 steps. First, we fit a propensity model of ICU admission (using decision trees and gradient boosted decision trees) using the entire surgical cohort. Then, among patients admitted to the ICU we fit a similar model with the response an indicator variable of having any CAM-ICU assessments.

The ICU admission model had relatively good predictive performance (AUROC 0.941). A histogram of the fitted values of the ICU-admission model (eFigure 3) shows that while a substantial fraction of ICU patients have high propensity to admission, about half lay in an overlapping region with non-admitted patients. Model diagnostics showed that surgical procedure (CCS category), emergency surgery, and ASA-PS explained the majority of the fitted propensity (R^2 = 82%). Examination of the fitted model showed that OSA status was never used as a decision tree node. However, because of its correlation to other variables, OSA diagnosis was correlated with fitted propensity to admission (regression coefficient 0.37, SE 0.04 on log-odds scale) but explained only 0.7% of the variation in propensity. In the sensitivity analyses below, the propensity model above is used to select unadmitted patients to serve as additional controls.

© 2020 King CR et al. *JAMA Network Open.*

eFigure 3. Histogram of Propensity to ICU Admission in Admitted and Unadmitted Patients

X-axis: log-odds of propensity to ICU admission from gradient boosted decision tree model. Y-axis: counts. Blue: admitted to ICU. Red: not admitted to ICU.

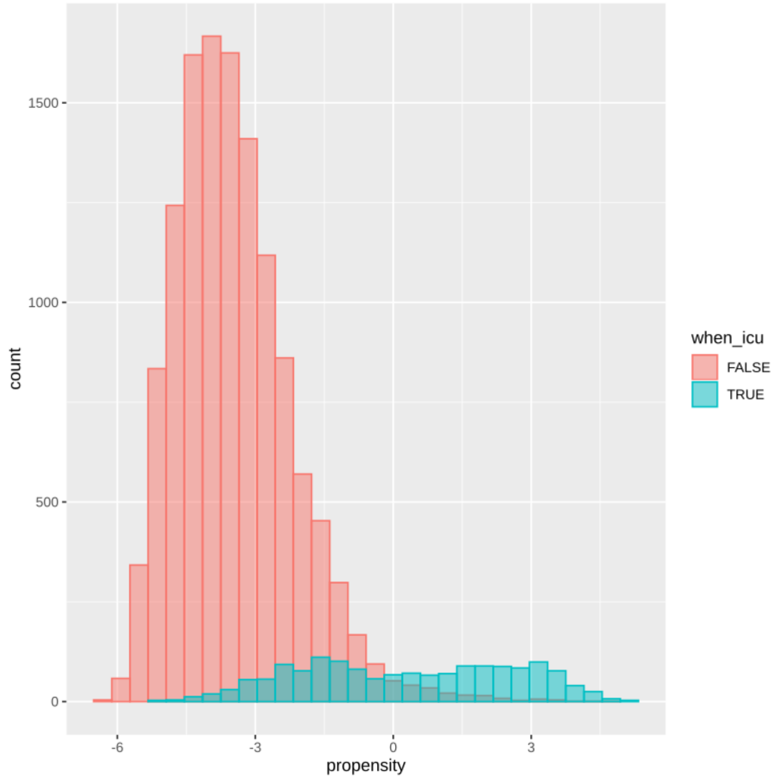

The assessment model had relatively poor predictive performance (AUROC 0.619). Correlates of never-assessment were ASA-PS 5 (9% vs 2%), length of ICU stay <= 24 hours (32% vs 15%), and in-hospital mortality (24% vs 6%). Using a model on the assessed patients (excluding OSA status) applied to the never assessed patients, the predicted probabilities for delirium had a mean of 51% and (0.1, 0.25, 0.5, 0.75, 0.9) quantiles of [0.23 0.34 0.51 0.68 0.79]. Using a hold-out sample of assessed patients, the mean probability was 47% and quantiles [0.20 0.30 0.44 0.64 0.77]. These suggest that never-assessed patients were enriched in both moribound individuals, who may have not been assessed because of a lack of adequate mental status, and individuals with low risk of delirium and very short stays, but that unassessed patients were not systematically very different in delirium risk. The raw odds-ratio for never-assessment by OSA is 1.16 (95% CI 1.00 to 1.35, p=0.046). OSA status was modestly correlated with fitted propensity (regression coefficient 0.20 SE .03 on log-odds scale) with very little explained variation (R^2=1.9%). It was the 28th most influential predictor by mean absolute Shapely value. eFigure 4 plots a similar propensity histogram to eFigure 3. Because all non-assessed patients were in a broadly overlapping region of propensity with assessed patients, no matching procedure was used for the below sensitivity analysis.

© 2020 King CR et al. *JAMA Network Open.*

eFigure 4. Histogram of Propensity to CAM-ICU Assessment Among ICU Patients in Assessed and Unassessed Patients

X-axis: log-odds of propensity to CAM-ICU performance from gradient boosted decision tree model. Y-axis: counts. Blue: assessed. Red: not assessed.

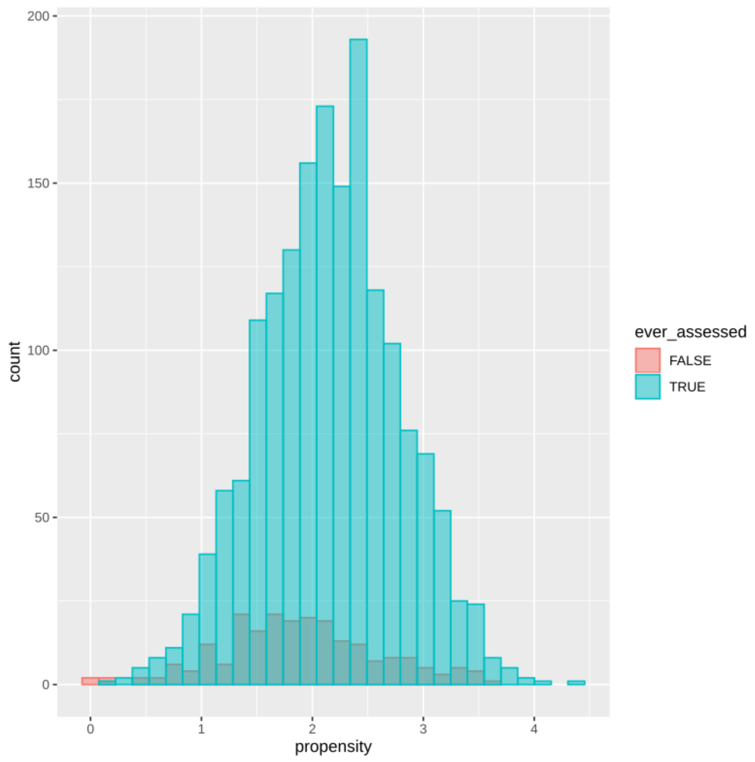

© 2020 King CR et al. *JAMA Network Open.*

eTable 3. Covariates by CAM-ICU Assessment Status and OSA Status

Among those admitted to eligible ICUs and without pre-admission >= 6 days. See Table 1 for definitions.

	OSA -		OSA +	
	No CAM	Any CAM	No CAM	Any CAM
	(n=893)	(n=6417)	(n=259)	(n=2161)
Female Sex				
Yes	393 (44.0%)	2827 (44.1%)	77 (29.7%)	719 (33.3%)
No	500 (56.0%)	3590 (55.9%)	182 (70.3%)	1442 (66.7%)
Surgery type				
Operations on the cardiovascular system	410 (45.9%)	2681 (41.8%)	135 (52.1%)	1000 (46.3%)
Operations on the digestive system	130 (14.6%)	1240 (19.3%)	35 (13.5%)	343 (15.9%)

© 2020 King CR et al. *JAMA Network Open.*

Operations on the female genital organs	12 (1.3%)	72 (1.1%)	3 (1.2%)	21 (1.0%)
Operations on the hemic and lymphatic system	7 (0.8%)	23 (0.4%)	3 (1.2%)	10 (0.5%)
Operations on the integumentary system	11 (1.2%)	104 (1.6%)	1 (0.4%)	37 (1.7%)
Operations on the male genital organs	2 (0.2%)	15 (0.2%)	4 (1.5%)	11 (0.5%)
Operations on the musculoskeletal system	115 (12.9%)	874 (13.6%)	25 (9.7%)	335 (15.5%)
Operations on the nervous system	21 (2.4%)	138 (2.2%)	2 (0.8%)	36 (1.7%)

© 2020 King CR et al. *JAMA Network Open.*

Operations on the nose; mouth; and pharynx	8 (0.9%)	39 (0.6%)	1 (0.4%)	13 (0.6%)
Operations on the respiratory system	70 (7.8%)	283 (4.4%)	17 (6.6%)	84 (3.9%)
Operations on the urinary system	17 (1.9%)	175 (2.7%)	8 (3.1%)	60 (2.8%)
Other	90 (10.1%)	773 (12.0%)	25 (9.7%)	211 (9.8%)
ASA				
1	9 (1.0%)	67 (1.0%)	0 (0%)	4 (0.2%)
2	118 (13.2%)	860 (13.4%)	18 (6.9%)	187 (8.7%)
3	313 (35.1%)	2488 (38.8%)	91 (35.1%)	964 (44.6%)
4	372 (41.7%)	2841 (44.3%)	132 (51.0%)	986 (45.6%)
5	81 (9.1%)	161 (2.5%)	18 (6.9%)	20 (0.9%)
RACE				

© 2020 King CR et al. *JAMA Network Open.*

Unknown	34 (3.8%)	214 (3.3%)	0 (0%)	46 (2.1%)
Black	158 (17.7%)	1078 (16.8%)	43 (16.6%)	270 (12.5%)
White	655 (73.3%)	4857 (75.7%)	204 (78.8%)	1805 (83.5%)
Other	46 (5.2%)	268 (4.2%)	12 (4.6%)	40 (1.9%)
CAD				
Yes	218 (24.4%)	1520 (23.7%)	100 (38.6%)	815 (37.7%)
No	675 (75.6%)	4897 (76.3%)	159 (61.4%)	1346 (62.3%)
Atrial fib				
Yes	47 (5.3%)	450 (7.0%)	59 (22.8%)	290 (13.4%)
No	846 (94.7%)	5967 (93.0%)	200 (77.2%)	1871 (86.6%)
COPD				
Yes	97 (10.9%)	746 (11.6%)	63 (24.3%)	441 (20.4%)
No	796 (89.1%)	5671 (88.4%)	196 (75.7%)	1720 (79.6%)
CKD				

© 2020 King CR et al. *JAMA Network Open.*

Yes	126 (14.1%)	817 (12.7%)	80 (30.9%)	497 (23.0%)
No	767 (85.9%)	5600 (87.3%)	179 (69.1%)	1664 (77.0%)
Dementia				
Yes	7 (0.8%)	32 (0.5%)	3 (1.2%)	14 (0.6%)
No	886 (99.2%)	6385 (99.5%)	256 (98.8%)	2147 (99.4%)
age (years)				
Mean (SD)	57 (17)	58 (16)	60 (13)	62 (12)
Median [Q1, Q3]	60 [47, 69]	60 [48, 69]	60 [53, 69]	63 [55, 70]
BMI (mk/kg**2)				
Mean (SD)	28 (7.2)	28 (6.7)	34 (8.5)	34 (8.5)
Median [Q1, Q3]	27 [23, 31]	27 [23, 31]	33 [28, 38]	32 [28, 38]
Missing	114 (12.8%)	745 (11.6%)	7 (2.7%)	62 (2.9%)

© 2020 King CR et al. *JAMA Network Open.*

HTN				
Yes	348 (39.0%)	2704 (42.1%)	193 (74.5%)	1513 (70.0%)
No	545 (61.0%)	3713 (57.9%)	66 (25.5%)	648 (30.0%)
CCI				
Mean (SD)	2.7 (2.4)	2.7 (2.3)	3.3 (2.4)	3.3 (2.3)
Median [Q1, Q3]	2.0 [1.0, 4.0]	2.0 [1.0, 4.0]	3.0 [2.0, 5.0]	3.0 [2.0, 5.0]
Missing	14 (1.6%)	67 (1.0%)	3 (1.2%)	19 (0.9%)
Risk strat index 1				
Mean (SD)	-0.42 (0.46)	-0.44 (0.48)	-0.41 (0.49)	-0.46 (0.49)
Median [Q1, Q3]	-0.44 [-0.66, -0.20]	-0.44 [-0.66, -0.21]	-0.44 [-0.66, 0.18]	-0.46 [-0.81, -0.20]
Missing	25 (2.8%)	238 (3.7%)	7 (2.7%)	86 (4.0%)
ZTCA poverty				
Mean (SD)	16 (10)	17 (9.8)	15 (8.8)	16 (9.0)

© 2020 King CR et al. *JAMA Network Open.*

Median [Q1, Q3]	16 [8.7, 21]	15 [9.0, 22]	14 [7.8, 21]	15 [9.0, 21]
Missing	349 (39.1%)	2329 (36.3%)	112 (43.2%)	736 (34.1%)
ZTCA employment				
Mean (SD)	0.92 (0.046)	0.92 (0.049)	0.92 (0.054)	0.92 (0.042)
Median [Q1, Q3]	0.93 [0.91, 0.95]	0.93 [0.91, 0.95]	0.94 [0.90, 0.95]	0.93 [0.91, 0.95]
Missing	349 (39.1%)	2329 (36.3%)	112 (43.2%)	736 (34.1%)
ZTCA housing				
Mean (SD)	11 (7.6)	11 (7.3)	11 (8.0)	11 (6.6)
Median [Q1, Q3]	9.4 [6.8, 13]	9.5 [6.9, 13]	9.6 [6.4, 13]	9.3 [7.1, 12]
Missing	349 (39.1%)	2329 (36.3%)	112 (43.2%)	736 (34.1%)
Postop benzodiazepines				
Yes	262 (29.3%)	1886 (29.4%)	80 (30.9%)	580 (26.8%)

© 2020 King CR et al. *JAMA Network Open.*

No	631 (70.7%)	4531 (70.6%)	179 (69.1%)	1581 (73.2%)
Postop sedation				
Yes	130 (14.6%)	1547 (24.1%)	34 (13.1%)	492 (22.8%)
No	763 (85.4%)	4870 (75.9%)	225 (86.9%)	1669 (77.2%)
Postop intubation				
Yes	481 (53.9%)	4034 (62.9%)	146 (56.4%)	1418 (65.6%)
No	412 (46.1%)	2383 (37.1%)	113 (43.6%)	743 (34.4%)
Postop NIPPV				
Yes	82 (9.2%)	1248 (19.4%)	70 (27.0%)	803 (37.2%)
No	811 (90.8%)	5169 (80.6%)	189 (73.0%)	1358 (62.8%)
Intraop oral morphine equiv				
Mean (SD)	81 (63)	86 (60)	83 (60)	95 (63)
Median [Q1, Q3]	68 [38, 110]	75 [38, 120]	68 [38, 120]	83 [46, 140]

© 2020 King CR et al. *JAMA Network Open.*

Pre and intraop midazolam dose				
Mean (SD)	2.7 (3.2)	2.6 (3.0)	2.3 (3.0)	2.5 (2.9)
Median [Q1, Q3]	2.0 [0.0, 5.0]	2.0 [0.0, 5.0]	2.0 [0.0, 5.0]	2.0 [0.0, 5.0]

© 2020 King CR et al. *JAMA Network Open.*

eAppendix 4. Propensity Score Generation and Primary Analysis

Within each imputation round, propensity scores were calculated, [4] and estimates of the average treatment effect (ATE) or average treatment effect on the treated (ATT) were computed (see below). Frequentist estimates were combined using Rubin's rules, and Bayesian posterior samples of treatment effects were concatenated into a single estimate. [5,6]

Propensity scores for the primary and secondary exposures were calculated using gradient boosted decision trees (xgboost library) within each imputation round, with all tuning parameters selected by 5-fold cross validation. All preoperative variables excluding OSA diagnosis, STOP-BANG total and questionnaire values, and neck measurement were included in the propensity model. Surgery was included as both the service category and single-level AHRQ CCS. We also experimented with propensity scores calculated by BART, and found them to be minimally different but much more time-consuming to calculate. Others have shown a close relationship between gradient boosted decision trees and BART point estimates. [7]

The primary analysis uses these scores as an additional adjusting variable, making the procedure "doubly robust" in that it can produce low-bias estimates if either the outcome model or exposure model is well-specified. [8] Although the practical performance of doubly robust Bayesian regression which includes propensity scores is well known, the theoretical rationale is usually not clear. [9] In the case of BART, which performs well without modification for causal inference in many circumstances, [10] it can be viewed as a re-parameterization which more easily captures selection bias based on perceived risks. [8,11] Propagation of uncertainty in propensity was not used as it has not been shown to improve performance. [9] Cross-validation using an 80% subsample was used to select hyperparameters. This strategy empirically has low bias and total error for causal effects compared to multiple comparators. [12] A histogram of propensity to OSA is shown in eFigure 5.

BART is a Bayesian procedure and therefore produces credible intervals instead of confidence intervals. The credible interval is the area of the posterior with the highest density containing 99% of the probability for the calculated quantity. Because it is non-parametric, the prior is over the functional form of fitted surfaces in the form of splitting probabilities for decision trees, and the "prior" for treatment effects is only indirectly induced. The meaning of the BART prior has been discussed extensively elsewhere. [10,11] While there are guarantees of concordance between credible intervals and confidence intervals only in some specialized cases, others have observed that BART estimated treatment effects have good frequentist properties as well. [12]

The association of PAP with delirium was analyzed within OSA + patients only. Patients without OSA (not eligible for PAP therapy) remained excluded even if they used BPAP or CPAP for respiratory failure. The "PAP effect" therefore compares "OSA +, PAP-" to "OSA +, PAP +" and no interaction term is required. Because the OSA-restricted sample size is small and because PAP adherence was so frequently missing, we also experimented with adding a "missing" factor level and a term for missing included in the model and with including OSA - patients in the model. "Non-adherent" remained the reference level, and reported effects are for the "adherent vs non-adherent" contrast. Inclusion of these unknown exposure level observations does not directly contribute to the estimate; however, they have an indirect effect of stabilizing the estimated effects of covariates. Because the PAP+ patients are a subset of OSA+, no interaction term is required.

During the preparation of the manuscript, Hahn and colleagues released the bcf package implementing their methods. However, bcf did not accommodate binary outcomes, so the presented results use a customized BART 1.7 validated on the examples of Hill (data not shown). [10] The optmatch package performed matching; mice performed imputation, and the margins package estimated marginal effects for generalized linear models.

© 2020 King CR et al. *JAMA Network Open.*

eFigure 5. Histogram of OSA Propensity Score by OSA Status in the Entire Surgical Population
X-axis: log-odds of propensity to OSA. Y-axis: counts. Blue: yes OSA. Red: no OSA.

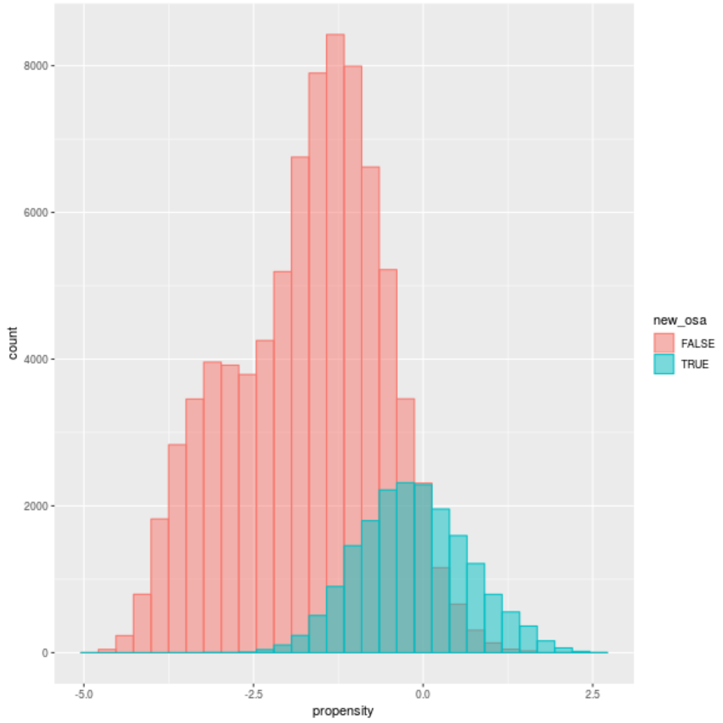

© 2020 King CR et al. *JAMA Network Open.*

eAppendix 5. Sensitivity Analyses and Alternative Analytic Approaches

We validated these calculations by comparison to other methods. These included propensity score matching with 1:1 optimal matching [13] using BART or logistic regression to generate scores instead of gradient boosting, adjustment for covariates in classic BART, classic BART plus propensity scores as a covariate, and logistic regression. We conducted several sensitivity analyses.

1) We performed imputation and estimated an alternative propensity score using the entire perioperative dataset with an indicator variable for non-ICU admission.
2) We restricted the analysis to only patients with pre-specified surgical service (an option field serving as a marker of a complete anesthesia assessment) or only patients whose evaluation was performed in preoperative clinic.
3) We added all patients otherwise meeting inclusion criteria but never CAM-ICU assessed as "negative," all as "positive," and with a random draw from fitted values from a model excluding OSA status.
4) We added patients otherwise meeting inclusion criteria but not admitted to the ICU with a "negative" value for delirium. These patients were selected using a nonparametric model of ICU admission (see above) and 1:1 matched to admitted patients with a similar propensity for admission (20 strata), the same OSA status, and the same CCS level-2 primary procedure code.
5) We excluded components of STOP-BANG (hypertension, gender, BMI, age) as covariates.
6) We imputed missing data without including outcomes as a covariable.
7) We defined the exposure as OSA *diagnosis* only (not using STOP-BANG).
8) We added potential mediators of an OSA-delirium relationship as covariates (intra- and postoperative benzodiazepine use, total intraoperative use opioid in oral morphine equivalents, postoperative ventilation, postoperative positive airway pressure use).
9) We evaluated a regression of STOP-BANG score versus postoperative delirium only among patients without an OSA diagnosis. Several of the covariates are components of STOP-BANG; the eAppendix 7 contains the effective contrasts being drawn when conditioning on BMI, age, sex, and history of hypertension. The effect of STOP-BANG was evaluated by comparison of fitted values counterfactually setting it to "0".

© 2020 King CR et al. *JAMA Network Open.*

eAppendix 6. Hyperparameters, Tuning, and Variable Importance

Duration of markov-chain monte carlo sampling was increased until Geweke diagnostics and autocorrelation-based effective sample sizes were found to be adequate. During tuning of OSA status prediction we used 20,000 posterior draws after a burn-in of 5,000 draws on a singly imputed dataset with an 80:20 cross validation split. During final propensity score calculation, we used 15,000 draws after a 5,000 burn in period for each of 30 imputed datasets. During PAP prediction tuning we used 50,000 posterior draws after a burn-in of 10,000. During BCF and BART prediction of delirium we used 30,000 posterior draws after a burn-in of 5,000 in each of 30 imputed datasets.

For the prediction of OSA status, cross-validation of AUROC selected the following BART parameters: k = 2.76, n_tree = 248. For the prediction of PAP adherence, k = 2.675, n_tree = 59. A sparse dirichlet prior made no meaningful improvement in AUROC (difference of .002) and substantially increased computing requirements. For predicting delirium with only the OSA propensity score, the optimal k was 3.1 and n_tree was 15. For predicting delirium with BCF k = 2.15 and n_tree = 231, ntree_treated = 41. For pure BART predicting delirium k = 2.5, n_tree = 162.

We observed the results to not depend strongly on these parameters. We kept the power and base split probabilities at their default (2, 0.95). We observed minimal dependence of the effect estimates on hyperparameters. For example, using default parameters (k=2, ntree=200 or ntree=50) did not change the point estimate for the effect of OSA by more than 0.01 but did change credible interval widths increasing them by up to 0.03.

Predictor relevance was assessed using loss in predictive accuracy when permuting the predictor, coverage probabilities, improvement in splitting, and mean absolute shapely value. [14]

For the matched analysis, we used 1:1 optimal matching with a caliper of 1 on the logistic scale. In order to successfully match all exposed, in the analysis of PAP adherence the caliper was increased to 1.5.

Propensity-adjusted sample comparisons and standardized differences were computed using inverse propensity weights.

© 2020 King CR et al. *JAMA Network Open.*

eAppendix 7. STOP-BANG Contrasts

Adjusting for covariates always explains away some of the exposure, but in the case where the exposure is a direct function of some covariates, the implicit contrast being drawn by the observed effect of the residual exposure is somewhat complicated.

Take the high-risk STOP-BANG screens as the clearest example. The goal is to compare outcome rates between patients with high risk screens and those without. Elements of the screen (PBAG=BMI, gender, hypertension, age) also have likely direct effects on the outcomes, and so we adjust them as if they were confounders. If we then say STOP-BANG > 4 is the threshold, the net contrasts being drawn are then within strata of PBAG using the number of other factors (SNOT = snoring, tiredness, observed, neck circumference)

PBAG = 0 : discard data (there are no STOP-BANG > 4)
PBAG = 1 : compare SNOT = 4 to SNOT <= 3
PBAG = 2 : compare SNOT > 2 to SNOT <= 2
PBAG = 3 : compare SNOT > 1 to SNOT <= 1
PBAG = 4 : compare SNOT > 0 to SNOT = 0

With the more complicated scheme that is now recommended the contrast is more convoluted (add the letter H for bicarb):

PBAG = 4 : compare SNOT > 0 to SNOT = 0 (H irrelevant)
{BG = 2 and P=0 and A=1} : compare {STO > 1} or {STO =1 and N} to {STO = 0} or {STO = 1 and N =0} (H irrelevant)
{BG = 2,1 and P=1 any A} : compare STO > 0 to STO = 0 (NH irrelevant)
{BG = 2 and P=0 and A=0} : compare STO > 1 to STO <= 1 (NH irrelevant)
{BG = 1 and P =0 any A} : compare STO > 1 to STO <= 1 (NH irrelevant)
{BG = 0 and P =1 and A =0} : compare {HN > 0 and STO >0} to {HN>0 and STO =0} and {HN=0}
{BG = 0 and P =1 and A =1} : compare {HN > 0 and STO >0} or {HN=0 and STO =3} to {HN>0 and STO =0} and {HN=0 and STO < 3}
{BG = 0 and P =0 any A} : compare {HN > 0 and STO >1} to {HN>0 and STO <=1} and {HN=0}

© 2020 King CR et al. *JAMA Network Open.*

eTable 4. Covariance and Marginal Distribution of STOP-BANG Elements
Rounded to 2 digits.
Panel A: Among all preop evaluated patients:

	mean	Pressure	Snore	Observed	Tired	Neck	BMI	Gender	Age
Pressure	0.45	0.44	0.25	0.03	0.01	0.02	0.03	0.02	0.02
Snore	0.33	0.33	0.03	0.22	0.03	0.04	0.03	0.03	0.02
Observed	0.07	0.07	0.01	0.03	0.06	0.02	0.01	0.01	0.01
Tired	0.30	0.3	0.02	0.04	0.02	0.21	0.01	0.01	-0.01
Neck	0.22	0.22	0.03	0.03	0.01	0.01	0.17	0.06	0.05
BMI	0.20	0.2	0.02	0.03	0.01	0.01	0.06	0.16	-0.02
Gender	0.48	0.48	0.02	0.02	0.01	-0.01	0.05	-0.02	0.25
Age	0.66	0.64	0.09	0.02	0	0.01	0.01	-0.01	0.02

Panel B: Among ICU patients. Final columns odds-ratio predicting delirium with 95% confidence interval from univariate GLM.

	mean	P	S	O	T	N	B	G	A	OR	95% ci
P	0.58	0.59	0.24	0.03	0.01	0.02	0.03	0.02	0.02	0.04	1.07
S	0.37	0.37	0.03	0.23	0.04	0.03	0.04	0.03	0.02	0.01	0.99
O	0.09	0.09	0.01	0.04	0.08	0.02	0.01	0	0.01	0	1.03
T	0.37	0.37	0.02	0.03	0.02	0.23	0	0	-0.02	0	1.04
N	0.24	0.25	0.03	0.04	0.01	0	0.19	0.08	0.05	-0.01	0.98
B	0.2	0.19	0.02	0.03	0	0	0.08	0.15	-0.01	-0.01	1.01
G	0.57	0.58	0.02	0.02	0.01	-0.02	0.05	-0.01	0.24	0	1.01
A	0.80	0.8	0.04	0.01	0	0	-0.01	-0.01	0	0.16	1.05

© 2020 King CR et al. *JAMA Network Open.*

eTable 5. Prevalence of OSA Diagnosis and Rate of Missing STOP-BANG Criteria by Surgical Category

Panel A: Patients not admitted to the ICU. # = number of cases, all other rounded to tenths of a percent. Age and gender never missing.

Surgical service	#	osa	s	t	o	p	b	n	Total
cardsurg	1056	26	54.3	53.2	57.9	52.6	0.9	79.3	23.1
ent	2942	16.5	22.1	18.3	21.5	18.2	0.1	13.9	3.9
general	4574	18.3	24.9	21.5	25.4	20.8	0.3	18.8	3.7
gynecology	3871	14.1	16.8	12.8	17.1	12.2	0.2	6.6	0.5
neurosurgery	3232	25.2	26.4	23.8	26.8	23.2	0.2	18.8	3.4
orthopedic	5474	25.9	27.3	23.9	27.2	23.4	0.3	22.3	2.6
thoracic	1957	15.4	22.5	20.4	22.7	19.8	0.1	19.1	4.8
transplant	539	22.3	26.2	23.4	27.1	23.2	0.2	19.5	1.9
unknown	11448	4.2	44.3	40.3	43.4	39.7	8	68.3	15.5
urology	3948	21.6	23.2	19.2	22.8	18.5	0.2	8.9	0.4
vascular	1457	16.3	23.5	21.1	25.5	19.7	0.1	25.3	2.3
other	713	20.1	34.4	30.3	35.1	29.7	0.1	39.8	8.1

Panel B: Patients admitted to the ICU

Surgical service	#	osa	s	t	o	p	b	n	Total
cardsurg	1559	26.7	47.5	46.9	51.6	46	1.2	69	15.4
general	473	24.5	28.5	25.6	30.4	25.2	1.1	22.8	6.1
orthopedic	337	35	41.8	39.2	41.5	38.3	0.9	35.6	14.2
thoracic	212	16	34.4	31.6	35.8	31.6	0.5	38.2	20.8
unknown	1267	9.7	72.7	69.5	71.6	69.1	28.7	85.2	53.8
vascular	234	22.2	39.7	37.2	38.9	34.6	1.3	33.3	9
other	584	23.6	36	32.5	37.3	31.7	0.3	34.1	7.7

© 2020 King CR et al. *JAMA Network Open.*

eTable 6. Comparison of Effect Estimates From Sensitivity Analyses

"-0.00" and "0.00" are negative and positive numbers rounding to 0 at 2 digits. If unstated analytic method doubly-robust BCF, number of imputations = 30. ATE= average treatment effect, ATT=average treatment effect on the treated, AME=average marginal effect.

Panel A: Sensitivity analyses for OSA effect.

Analysis	Target	Estimate	99% LB	99% UB
Matched non-ICU -> negative delirium	ATE	0.01	-0.02	0.04
Matched non-ICU -> negative delirium	ATT	0.01	-0.02	0.04
Missing CAM = 0	ATE	-0.01	-0.04	0.03
Missing CAM = 0	ATT	-0.01	-0.04	0.03
Missing CAM = 1	ATE	-0.01	-0.04	0.03
Missing CAM = 1	ATT	-0.01	-0.04	0.03
Missing CAM ~ prob	ATE	-0.00	-0.04	0.03
Missing CAM ~ prob	ATT	-0.01	-0.04	0.03
Mediators included	ATE	-0.03	-0.06	0.01
Mediators included	ATT	-0.03	-0.06	0.01
No post-exposure in impute	ATE	-0.00	-0.04	0.03
No post-exposure in impute	ATT	-0.01	-0.04	0.03
Diagnosed OSA only	ATE	-0.01	-0.05	0.03
Diagnosed OSA only	ATT	-0.01	-0.05	0.02
Pre-screening only	ATE	0.02	-0.04	0.08
Pre-screening only	ATT	0.02	-0.05	0.08
Post-Screening Only	ATE	-0.02	-0.07	0.02
Post-Screening Only	ATT	-0.02	-0.07	0.02
No unk service	ATE	-0.01	-0.05	0.03
No unk service	ATT	-0.01	-0.05	0.03
Anest Clinic only	ATE	0.02	-0.03	0.07
Anest Clinic only	ATT	0.02	-0.03	0.07
Analysis plan full model	ATT	-0.01	-0.05	0.03

© 2020 King CR et al. *JAMA Network Open.*

Unadjusted - BCF	ATE	-0.03	-0.07	-0.00
Unadjusted - BCF	ATT	-0.04	-0.07	0.00
BCF Propensity only	ATE	0.00	-0.04	0.05
BCF Propensity only	ATT	-0.00	-0.04	0.04
Full model	ATE	-0.01	-0.04	0.03
Full model	ATT	-0.01	-0.04	0.03
BCF No propensity	ATE	-0.01	-0.04	0.03
BCF No propensity	ATT	-0.01	-0.04	0.03
BART with propensity and covariables	ATE	-0.00	-0.03	0.02
BART with covariables	ATE	-0.00	-0.03	c
BCF limited covariables	ATE	-0.01	-0.14	0.12
BCF limited covariables	ATT	-0.01	-0.14	0.12
BART global propensity	ATE	-0.01	-0.03	0.03
Full model global propensity	ATE	-0.00	-0.03	0.04
BCF global propensity only	ATE	-0.00	-0.04	0.05
1 impute BCF propensity only	ATE	0.01	-0.04	0.04
1 impute BCF propensity only	ATT	0.01	-0.03	0.04
1 impute full model	ATE	0.01	-0.04	0.04
1 impute full model	ATT	0.01	-0.03	0.04
1 impute BART covariables only	ATE	-0.00	-0.02	0.02
1 impute BCF no propensity	ATE	-0.00	-0.03	-0.03
1 impute BCF no propensity	ATT	-0.00	-0.03	-0.03
1 impute BCF limited covariables	ATE	-0.00	-0.11	0.11
1 impute BART with propensity and covariables	ATE	-0.00	-0.02	0.02
1 impute BCF global propensity	ATE	0.01	-0.04	0.05
1 impute BCF global propensity	ATT	-0.01	-0.03	0.05
Propensity matched	ATT	-0.01	-0.05	0.04
Linear propensity matched	ATT	-0.00	-0.06	0.05

Logistic regression model	AME	-0.02	-0.07	0.03

Panel B: PAP effect models. Abbreviations as above

Unadjusted	ATT	0.00	-0.09	0.09
BCF Propensity only	ATE	-0.03	-0.12	0.07
BCF Propensity only	ATT	-0.02	-0.12	0.08
Full model	ATE	-0.00	-0.07	0.07
Full model	ATT	-0.00	-0.08	0.07
BCF No propensity	ATE	-0.00	-0.08	0.08
BCF No propensity	ATT	-0.00	-0.08	0.07
BART with propensity and covariables	ATE	-0.00	-0.07	0.06
BART with covariables	ATE	-0.00	-0.06	0.07
PAP linear model	AME	0.01	-0.06	0.08
PAP linear propensity match	ATT	-0.02	-0.16	0.11
PAP no augmentation	ATE	0.00	-0.09	0.08
PAP no augment propensity only	ATE	0.00	-0.09	0.08
PAP full propensity match	ATT	-0.02	-0.13	0.10

Panel C: STOP-BANG effects, computed among those without an OSA diagnosis.

STOP-BANG BART	AME	0.00	-0.04	0.06
STOP-BANG logistic model	AME	0.00	-0.02	0.02
STOP-BANG logistic model	lOR	0.01	-0.08	0.11

© 2020 King CR et al. *JAMA Network Open.*

eTable 7. Logistic Regression Coefficients

Panel A: modeling CAM-ICU+. CCS categories omitted from model.

	**delirium ever**		
Predictors	*Odds Ratios*	*CI*	*p*
Historical preop only	1.11	0.96 – 1.30	0.163
Female sex	1.03	0.93 – 1.15	0.566
FUNCTIONAL CAPACITY	1.29	1.17 – 1.42	**<0.001**
History of HTN	0.94	0.82 – 1.07	0.330
History of CAD	0.83	0.71 – 0.97	**0.016**
PRIOR MI	1.21	1.01 – 1.44	**0.038**
History of CHF	0.94	0.80 – 1.10	0.428
Diastolic Dysfunction	1.05	0.87 – 1.26	0.619
LVEF	1.06	1.01 – 1.11	**0.031**
History of Aortic stenosis	0.91	0.82 – 1.01	0.084
History of AFIB	0.97	0.81 – 1.16	0.728
PPM ICD implanted	0.98	0.81 – 1.20	0.871
History of TIA or STROKE	0.94	0.72 – 1.23	0.658
History of PAD	1.36	1.08 – 1.70	**0.008**

© 2020 King CR et al. *JAMA Network Open.*

History of DVT	1.28	1.04 – 1.57	**0.020**
History of PE	0.98	0.72 – 1.32	0.883
History of DM	1.07	0.92 – 1.23	0.389
Outpatient Insulin use	1.09	0.89 – 1.34	0.411
History of CKD	1.28	1.09 – 1.50	**0.003**
History of Dialysis	1.00	0.86 – 1.18	0.958
History of Pulm Htn	1.12	0.95 – 1.31	0.178
History of COPD	1.10	0.95 – 1.27	0.216
History of ASTHMA	1.00	0.82 – 1.22	0.999
History of CIRRHOSIS	1.24	0.90 – 1.71	0.196
History of CANCER	0.93	0.81 – 1.07	0.295
History of GERD	0.92	0.82 – 1.04	0.192
History of ANEMIA	1.06	0.94 – 1.21	0.347
History of COOMBS POSITIVE	0.64	0.45 – 0.93	**0.018**
History of DEMENTIA	2.87	1.40 – 5.90	**0.004**
SMOKING EVER	1.07	0.96 – 1.19	0.240
PreOp Diastolic	0.99	0.93 – 1.05	0.657
PreOp Systolic	1.02	0.96 – 1.08	0.582

© 2020 King CR et al. *JAMA Network Open.*

PreOp SpO2	1.01	0.96 – 1.06	0.752
PreOp HR	1.22	1.16 – 1.28	**<0.001**
age	1.26	1.18 – 1.34	**<0.001**
ASA2	1.14	0.65 – 1.99	0.651
ASA3	1.55	0.89 – 2.71	0.119
ASA4	2.73	1.56 – 4.77	**<0.001**
ASA5	4.55	2.33 – 8.88	**<0.001**
emergency	1.47	1.25 – 1.74	**<0.001**
BMI	0.95	0.90 – 1.00	0.057
RACE: Black	1.14	0.94 – 1.39	0.192
RACE: Other	1.35	1.10 – 1.65	**0.004**
Assessment Type: OTHER	1.40	1.15 – 1.70	**0.001**
Assessment Type: Inpatient	1.52	1.30 – 1.78	**<0.001**
Surg_Type: UNKNOWN	1.37	1.10 – 1.71	**0.005**
Surg_Type: GENERAL	1.26	1.02 – 1.57	**0.032**
Surg_Type: ORTHOPEDIC	1.95	1.54 – 2.46	**<0.001**
Surg_Type: THORACIC	1.04	0.80 – 1.34	0.788

© 2020 King CR et al. *JAMA Network Open.*

Surg_Type: VASCULAR	1.00	0.76 – 1.32	0.987
Surg_Type: UROLOGY	1.55	1.09 – 2.20	**0.015**
Surg_Type: OTHER	1.79	1.27 – 2.51	**0.001**
Surg_Type: NEUROSURGERY	2.49	1.74 – 3.58	**<0.001**
Surg_Type: TRANSPLANT	1.12	0.74 – 1.69	0.587
Surg_Type: ENT	1.06	0.72 – 1.56	0.765
Surg_Type: GYNECOLOGY	2.07	1.31 – 3.27	**0.002**
OSA+Screen	1.02	0.90 – 1.15	0.749
Primary procedure risk strat index	1.12	1.00 – 1.26	0.051
Secondary procedure risk strat index	1.34	1.19 – 1.50	**<0.001**
ZTCA population	1.00	1.00 – 1.00	0.386
ZTCA urban	1.00	0.99 – 1.02	0.623
ZTCA black_percent	1.00	1.00 – 1.00	0.971
ZTCA asian_percent	1.00	0.97 – 1.03	0.957
ZTCA hispanic_percent	1.01	1.00 – 1.02	0.184
ZTCA vacant_housing	1.01	1.01 – 1.02	**0.001**

© 2020 King CR et al. *JAMA Network Open.*

ZTCA employed_lodds	1.08	0.97 – 1.21	0.165
ZTCA poverty_fraction	1.00	0.99 – 1.01	0.801
ZTCA ed_less_hs	0.31	0.09 – 1.07	0.063
ZTCA ed_hs	2.84	1.25 – 6.43	**0.013**
case_year2013	0.90	0.70 – 1.16	0.435
case_year2014	0.83	0.64 – 1.07	0.148
case_year2015	0.69	0.54 – 0.90	**0.006**
case_year2016	0.69	0.53 – 0.90	**0.007**
Observations	7792		
R^2 Tjur	0.126		

Panel B: Modeling OSA

	**All periop**			**ICU_Admit**		
Predictors	*Odds Ratios*	*CI*	*p*	*Odds Ratios*	*CI*	*p*
Historical preop only	1.06	0.99 – 1.14	0.081	1.19	0.99 – 1.42	0.066
Female sex	0.34	0.33 – 0.36	**<0.001**	0.39	0.34 – 0.44	**<0.001**

© 2020 King CR et al. *JAMA Network Open.*

FUNCTIONAL CAPACITY	1.18	1.14 – 1.23	**<0.001**	1.03	0.91 – 1.15	0.681
History of HTN	1.84	1.76 – 1.94	**<0.001**	1.50	1.29 – 1.74	**<0.001**
History of CAD	0.93	0.86 – 1.01	0.072	0.94	0.79 – 1.12	0.500
History of PRIOR MI	1.06	0.96 – 1.16	0.251	0.94	0.77 – 1.14	0.536
History of CHF	1.09	0.99 – 1.19	0.078	0.97	0.81 – 1.17	0.758
History of Diastolic Dysfunction	1.16	1.03 – 1.29	**0.010**	1.14	0.92 – 1.40	0.233
LVEF	1.04	1.01 – 1.08	**0.011**	1.08	1.02 – 1.15	**0.013**
History of Aortic stenosis	0.93	0.85 – 1.02	0.144	1.00	0.89 – 1.13	0.962
History of AFIB	1.21	1.10 – 1.32	**<0.001**	1.20	0.99 – 1.46	0.059
History of PPM_ICD	1.29	1.15 – 1.44	**<0.001**	1.54	1.24 – 1.91	**<0.001**
History of TIA or STROKE	1.11	0.99 – 1.25	0.069	0.83	0.61 – 1.11	0.205
History of PAD	0.99	0.89 – 1.10	0.837	0.95	0.74 – 1.21	0.663
History of DVT	0.98	0.90 – 1.06	0.557	1.02	0.81 – 1.29	0.840

© 2020 King CR et al. *JAMA Network Open.*

History of PE	1.10	0.97 – 1.24	0.157	1.08	0.77 – 1.51	0.667
History of DM	1.35	1.27 – 1.43	**<0.001**	1.24	1.05 – 1.45	**0.009**
Outpatient Insulin use	1.07	0.98 – 1.16	0.153	1.05	0.83 – 1.32	0.690
History of CKD	1.03	0.96 – 1.10	0.465	1.17	0.98 – 1.40	0.077
History of Dialysis	1.10	1.03 – 1.19	**0.007**	1.04	0.87 – 1.25	0.671
History of Pulm Htn	1.21	1.10 – 1.33	**<0.001**	1.43	1.19 – 1.71	**<0.001**
History of COPD	1.24	1.16 – 1.33	**<0.001**	1.52	1.29 – 1.80	**<0.001**
History of ASTHMA	1.45	1.36 – 1.55	**<0.001**	1.30	1.03 – 1.63	**0.024**
History of CIRRHOSIS	0.84	0.70 – 0.99	**0.042**	0.95	0.66 – 1.37	0.789
History of CANCER	0.92	0.88 – 0.97	**0.002**	0.93	0.79 – 1.09	0.354
History of GERD	1.40	1.34 – 1.46	**<0.001**	1.46	1.28 – 1.66	**<0.001**
History of ANEMIA	0.97	0.92 – 1.02	0.280	1.11	0.96 – 1.29	0.166
History of COOMBS POSITIVE	1.06	0.90 – 1.24	0.482	0.99	0.65 – 1.51	0.965

© 2020 King CR et al. *JAMA Network Open.*

History of DEMENTIA	0.84	0.66 – 1.07	0.150	1.46	0.72 – 2.94	0.291
SMOKING_EVER	1.17	1.12 – 1.23	**<0.001**	1.13	0.99 – 1.29	0.064
PreOp Diastolic	1.04	1.01 – 1.07	**0.012**	1.04	0.97 – 1.11	0.302
PreOp Systolic	0.93	0.90 – 0.95	**<0.001**	0.94	0.88 – 1.01	0.089
PreOp SpO2	0.93	0.91 – 0.95	**<0.001**	0.99	0.94 – 1.05	0.731
PreOp HR	0.95	0.93 – 0.98	**<0.001**	0.92	0.87 – 0.98	**0.008**
age	1.41	1.37 – 1.46	**<0.001**	1.33	1.22 – 1.45	**<0.001**
ASA2	1.76	1.50 – 2.06	**<0.001**	1.39	0.48 – 4.03	0.540
ASA3	2.04	1.72 – 2.42	**<0.001**	1.59	0.55 – 4.57	0.393
ASA4	2.07	1.72 – 2.50	**<0.001**	1.53	0.53 – 4.42	0.435
ASA5	1.75	1.19 – 2.57	**0.004**	0.93	0.29 – 3.00	0.899
emergency	1.00	0.90 – 1.12	0.964	1.20	0.97 – 1.48	0.097
BMI	2.49	2.43 – 2.55	**<0.001**	2.66	2.48 – 2.85	**<0.001**

© 2020 King CR et al. *JAMA Network Open.*

RACE: Black	0.78	0.72 – 0.84	**<0.001**	0.90	0.70 – 1.15	0.383
RACE: Other	0.77	0.67 – 0.88	**<0.001**	0.74	0.56 – 0.98	**0.034**
RACE: Unknown	0.98	0.79 – 1.21	0.829			
RACE: Asian	0.71	0.53 – 0.97	**0.030**			
Assessment type:DPAP (holding area)	0.53	0.41 – 0.68	**<0.001**			
Assessment type:DPAP (on ward)	0.35	0.30 – 0.41	**<0.001**			
Assessment type: inpatient	0.50	0.46 – 0.54	**<0.001**	0.58	0.48 – 0.70	**<0.001**
Assessment type:OTHER	0.57	0.51 – 0.64	**<0.001**	0.45	0.35 – 0.58	**<0.001**
surgery type: ORTHOPEDIC	1.97	1.79 – 2.18	**<0.001**	2.08	1.59 – 2.72	**<0.001**
surgery type: GENERAL	1.23	1.11 – 1.37	**<0.001**	1.02	0.80 – 1.30	0.883
surgery type: UROLOGY	1.30	1.17 – 1.44	**<0.001**	0.59	0.40 – 0.88	**0.010**

© 2020 King CR et al. *JAMA Network Open.*

surgery type: GYNECOLOGY	1.02	0.90 – 1.14	0.776	0.84	0.48 – 1.50	0.561
surgery type: ENT	1.39	1.24 – 1.56	**<0.001**	1.29	0.83 – 1.99	0.251
surgery type: CARDSURG	1.41	1.23 – 1.62	**<0.001**			
surgery type: NEUROSURGERY	2.40	2.14 – 2.70	**<0.001**	1.55	1.03 – 2.32	**0.033**
surgery type: THORACIC	1.25	1.10 – 1.41	**<0.001**	1.01	0.74 – 1.37	0.958
surgery type: VASCULAR	1.17	1.02 – 1.34	**0.024**	1.00	0.74 – 1.35	0.978
surgery type: TRANSPLANT	1.40	1.16 – 1.70	**0.001**	0.75	0.45 – 1.25	0.268
surgery type: OTHER	1.73	1.41 – 2.11	**<0.001**	1.12	0.76 – 1.65	0.576
surgery type: PLASTIC	1.46	1.13 – 1.89	**0.003**			
Primary procedure risk strat index	0.97	0.94 – 1.01	0.193	0.93	0.81 – 1.07	0.297
Secondary procedure risk strat index	0.96	0.91 – 1.01	0.094	0.93	0.81 – 1.07	0.330

© 2020 King CR et al. *JAMA Network Open.*

ZTCA population	1.00	1.00 – 1.00	0.543	1.00	1.00 – 1.00	0.279
ZTCA urban	0.99	0.99 – 1.00	**0.027**	1.00	0.99 – 1.02	0.617
ZTCA black_percent	1.00	1.00 – 1.00	0.629	0.99	0.99 – 1.00	**0.028**
ZTCA asian_percent	1.00	0.99 – 1.01	0.625	1.00	0.97 – 1.03	0.943
ZTCA hispanic_percent	1.00	0.99 – 1.01	0.699	1.00	0.99 – 1.02	0.785
ZTCA vacant_housing	0.99	0.99 – 1.00	**<0.001**	0.99	0.98 – 1.00	0.247
ZTCA employed_lodds	0.92	0.88 – 0.97	**0.001**	0.85	0.74 – 0.97	**0.014**
ZTCA poverty_fraction	1.00	0.99 – 1.00	0.352	0.99	0.98 – 1.00	0.235
ZTCA ed_less_hs	0.61	0.35 – 1.06	0.081	3.23	0.74 – 14.14	0.119
ZTCA ed_hs	0.74	0.53 – 1.04	0.085	0.81	0.31 – 2.16	0.679
case_year2013	1.07	0.96 – 1.20	0.235	1.10	0.78 – 1.53	0.596
case_year2014	1.52	1.35 – 1.70	**<0.001**	1.50	1.07 – 2.12	**0.020**
case_year2015	1.82	1.62 – 2.05	**<0.001**	1.56	1.11 – 2.20	**0.010**

© 2020 King CR et al. *JAMA Network Open.*

case_year2016	1.75	1.55 – 1.97	**<0.001**	1.40	0.98 – 1.99	0.063
surgery type: UNKNOWN				0.92	0.70 – 1.20	0.534
Observations	72643			7792		
R^2 Tjur	0.298			0.271		

Panel C: Predicting PAP adherence

	**All periop**			**ICU_Admit**		
Predictors	*Odds Ratios*	*CI*	*p*	*Odds Ratios*	*CI*	*p*
Historical preop only	1.00	0.84 – 1.19	0.987	0.83	0.52 – 1.33	0.447
Female sex	0.74	0.66 – 0.84	**<0.001**	0.95	0.65 – 1.38	0.782
FUNCTIONAL CAPACITY	0.86	0.78 – 0.95	**0.004**	0.91	0.64 – 1.28	0.580
History of HTN	0.98	0.86 – 1.11	0.740	0.68	0.45 – 1.04	0.074
History of CAD	1.06	0.90 – 1.25	0.464	1.42	0.92 – 2.18	0.117
History of PRIOR MI	0.75	0.62 – 0.91	**0.003**	0.61	0.38 – 0.99	**0.043**
History of CHF	1.13	0.95 – 1.35	0.167	1.36	0.87 – 2.12	0.175

© 2020 King CR et al. *JAMA Network Open.*

History of Diastolic Dysfunction	1.05	0.85 – 1.31	0.640	1.23	0.77 – 1.97	0.393
LVEF	1.01	0.94 – 1.09	0.717	0.94	0.80 – 1.10	0.421
History of Aortic stenosis	0.78	0.63 – 0.96	**0.016**	1.00	0.74 – 1.36	0.975
History of AFIB	1.05	0.89 – 1.24	0.531	1.58	1.02 – 2.45	**0.042**
History of PPM_ICD	0.99	0.80 – 1.23	0.943	0.76	0.46 – 1.27	0.292
History of TIA or STROKE	0.74	0.58 – 0.93	**0.011**	1.18	0.56 – 2.47	0.661
History of PAD	0.73	0.58 – 0.93	**0.010**	0.81	0.43 – 1.52	0.502
History of DVT	0.87	0.73 – 1.04	0.122	0.92	0.55 – 1.53	0.744
History of PE	0.83	0.64 – 1.09	0.176	0.49	0.22 – 1.13	0.095
History of DM	0.95	0.84 – 1.07	0.403	0.79	0.55 – 1.15	0.218
Outpatient Insulin use	0.94	0.80 – 1.11	0.479	1.07	0.67 – 1.70	0.770
History of CKD	0.88	0.76 – 1.03	0.107	1.19	0.78 – 1.82	0.410
History of Dialysis	1.08	0.93 – 1.26	0.314	0.77	0.51 – 1.16	0.214

© 2020 King CR et al. *JAMA Network Open.*

History of Pulm Htn	1.03	0.86 – 1.23	0.748	1.13	0.74 – 1.71	0.571
History of COPD	1.02	0.88 – 1.18	0.774	1.35	0.93 – 1.97	0.119
History of ASTHMA	1.09	0.95 – 1.24	0.216	1.02	0.64 – 1.63	0.938
History of CIRRHOSIS	1.01	0.69 – 1.49	0.954	0.79	0.31 – 2.00	0.619
History of CANCER	0.96	0.86 – 1.08	0.481	1.04	0.70 – 1.55	0.847
History of GERD	0.94	0.85 – 1.04	0.256	0.95	0.69 – 1.31	0.757
History of ANEMIA	0.76	0.68 – 0.85	**<0.001**	0.92	0.63 – 1.33	0.645
History of COOMBS POSITIVE	0.94	0.65 – 1.34	0.719	0.96	0.37 – 2.45	0.929
History of DEMENTIA	1.17	0.64 – 2.12	0.613	1.83	0.37 – 8.98	0.454
SMOKING_EVER	0.84	0.75 – 0.94	**0.002**	0.76	0.53 – 1.08	0.126
PreOp Diastolic	0.95	0.89 – 1.02	0.146	1.06	0.88 – 1.29	0.540
PreOp Systolic	0.99	0.93 – 1.06	0.791	0.92	0.76 – 1.10	0.340
PreOp SpO2	0.97	0.92 – 1.01	0.165	0.93	0.80 – 1.08	0.340

© 2020 King CR et al. *JAMA Network Open.*

PreOp HR	1.00	0.94 – 1.07	0.961	1.01	0.84 – 1.20	0.949
age	1.26	1.16 – 1.38	**<0.001**	1.24	0.95 – 1.62	0.119
ASA2	1.15	0.48 – 2.73	0.759			
ASA3	1.11	0.46 – 2.67	0.817	0.87	0.41 – 1.83	0.708
ASA4	1.37	0.56 – 3.38	0.494	0.68	0.30 – 1.54	0.355
ASA5	1.20	0.30 – 4.75	0.795	0.48	0.06 – 3.91	0.495
emergency	0.72	0.50 – 1.03	0.074	0.85	0.44 – 1.65	0.636
BMI	1.47	1.39 – 1.55	**<0.001**	1.33	1.12 – 1.58	**0.001**
RACE: Black	0.69	0.57 – 0.83	**<0.001**	0.80	0.42 – 1.54	0.509
RACE: Other	0.95	0.63 – 1.44	0.816	0.53	0.23 – 1.25	0.145
RACE: Unknown	0.53	0.29 – 0.98	**0.042**			
RACE: Asian	1.22	0.50 – 2.99	0.666			
Assessment type:DPAP (holding area)	1.27	0.61 – 2.62	0.520			

© 2020 King CR et al. *JAMA Network Open.*

Assessment type:DPAP (on ward)	0.92	0.65 – 1.30	0.652			
Assessment type: inpatient	1.11	0.90 – 1.38	0.339	1.16	0.69 – 1.94	0.579
Assessment type:OTHER	0.56	0.32 – 0.99	**0.048**	0.47	0.22 – 0.97	**0.042**
Surg_Type: ORTHOPEDIC	1.00	0.69 – 1.45	0.998	0.64	0.33 – 1.24	0.184
Surg_Type: GENERAL	0.84	0.58 – 1.21	0.347	0.51	0.28 – 0.93	**0.028**
Surg_Type: UROLOGY	1.01	0.69 – 1.47	0.974	0.63	0.22 – 1.85	0.402
Surg_Type: GYNECOLOGY	0.73	0.49 – 1.09	0.124	0.47	0.12 – 1.87	0.284
Surg_Type: ENT	0.77	0.52 – 1.16	0.210	0.90	0.34 – 2.37	0.828
Surg_Type: CARDSURG	1.28	0.83 – 1.96	0.262			
Surg_Type: NEUROSURGERY	0.78	0.52 – 1.15	0.211	1.26	0.39 – 4.03	0.697
Surg_Type: THORACIC	1.14	0.76 – 1.72	0.527	0.87	0.40 – 1.90	0.730
Surg_Type: VASCULAR	1.02	0.66 – 1.57	0.927	0.94	0.43 – 2.04	0.871
Surg_Type: TRANSPLANT	0.81	0.49 – 1.36	0.428	0.65	0.17 – 2.45	0.525

Surg_Type: OTHER	0.88	0.52 – 1.50	0.644	1.97	0.71 – 5.44	0.193
Surg_Type: PLASTIC	1.98	1.01 – 3.90	**0.048**			
Primary procedure risk strat index	1.00	0.91 – 1.10	0.980	0.92	0.61 – 1.38	0.682
Secondary procedure risk strat index	0.93	0.82 – 1.05	0.229	0.76	0.51 – 1.14	0.183
ZTCA population	1.00	1.00 – 1.00	**0.009**	1.00	1.00 – 1.00	0.197
ZTCA urban	1.00	0.98 – 1.01	0.610	1.06	1.00 – 1.11	**0.032**
ZTCA black_percent	1.01	1.00 – 1.01	**0.007**	1.00	0.99 – 1.01	0.979
ZTCA asian_percent	0.98	0.95 – 1.01	0.161	1.03	0.92 – 1.15	0.654
ZTCA hispanic_percent	1.02	1.00 – 1.04	**0.038**	0.98	0.93 – 1.04	0.557
ZTCA vacant_housing	1.00	0.99 – 1.01	0.790	1.02	0.99 – 1.05	0.211
ZTCA employed_lodds	1.21	1.08 – 1.37	**0.002**	1.22	0.85 – 1.76	0.286
ZTCA poverty_fraction	1.00	0.99 – 1.01	0.723	0.98	0.96 – 1.01	0.265
ZTCA ed_less_hs	0.13	0.03 – 0.53	**0.004**	4.98	0.10 – 248.31	0.421

© 2020 King CR et al. *JAMA Network Open.*

ZTCA ed_hs	0.82	0.34 – 1.94	0.651	7.06	0.58 – 86.57	0.126
case_year2013	0.97	0.73 – 1.30	0.853	0.83	0.31 – 2.22	0.712
case_year2014	0.91	0.67 – 1.23	0.536	0.66	0.24 – 1.81	0.419
case_year2015	0.87	0.64 – 1.18	0.365	0.54	0.20 – 1.46	0.224
case_year2016	0.81	0.60 – 1.11	0.187	0.58	0.21 – 1.62	0.299
Surg_Type: UNKNOWN				0.68	0.24 – 1.94	0.474
Observations	7231			849		
R^2 Tjur	0.077			0.141		

© 2020 King CR et al. *JAMA Network Open.*

eTable 8. Variable Importance Metrics for Propensity Models
Panel A permutes each variable to simulate the null distribution and displays the change in out of sample AUROC resulting from the loss of information. "Delta" column normalized to out-of-sample full model. Not all variables always included in model, row omitted in that case. Large negative deltas indicate an important variable. Rows with positive delta (model performs better scrambling them) omitted.

Panel A
Out of Sample AUROC when permuting predictors, outcome of OSA and PAP adherence

OSA Model			PAP Model		
names	auroc	delta	names	auroc	delta
Baseline (in sample)	0.875	0.0003	Baseline (in sample)	0.679	0.0711
Baseline (out of sample)	0.875	0	Baseline (out of sample)	0.608	0
WEIGHT	0.762	-0.1121	BMI	0.552	-0.0559
before_screening	0.788	-0.0869	SEX	0.601	-0.0072
HTN	0.857	-0.0172	RACE	0.602	-0.0063
SEX	0.859	-0.0158	Age_at_DoS	0.604	-0.0038
Surg_Type	0.86	-0.0148	SMOKING_EVER	0.605	-0.0028
Age_at_DoS	0.864	-0.0106	ccs_factor	0.606	-0.0024
HEIGHT	0.866	-0.0083	WEIGHT	0.606	-0.0023
GERD	0.869	-0.0061	Surg_Type	0.606	-0.0022
ASA	0.871	-0.0039	Pain	0.606	-0.0018
DM	0.871	-0.0034	LVEF	0.606	-0.0017
ccs_factor	0.873	-0.0019	acs_poverty_fraction	0.607	-0.0014
RACE	0.873	-0.0018	acs_employed_fraction	0.607	-0.0011

© 2020 King CR et al. *JAMA Network Open.*

CANCER_HX	0.873	-0.0014	CKD	0.607	-0.0011
ASTHMA	0.873	-0.0014	PreOp_Diastolic	0.608	-0.0004
SMOKING_EVER	0.874	-0.0011	case_year	0.608	-0.0001
FUNCTIONAL_CAPACITY	0.874	-0.0008			
COPD	0.874	-0.0006			
CHF	0.874	-0.0005			
PHTN	0.874	-0.0004			
PPM_ICD	0.874	-0.0004			
CAD	0.874	-0.0004			
case_year	0.874	-0.0003			
AFIB	0.874	-0.0003			
CCI	0.874	-0.0002			
LVEF	0.875	-0.0001			
acs_ed_college	0.875	-0.0001			
CHF_Diastolic_Function	0.875	-0.0001			
acs_white_percent	0.875	-0.0001			
acs_ed_less_hs	0.875	-0.0001			
acs_black_percent	0.875	-0.0001			

Panel B
Influence measures from gradient boosted decision trees. Predicting PAP

© 2020 King CR et al. *JAMA Network Open.*

	Feature	Gain	Cover	Frequency	shap
1	WEIGHT	0.136	0.056	0.057	0.113
2	ed_less_hs	0.08	0.054	0.057	0.214
3	BMI	0.078	0.064	0.072	0.34
4	Age	0.058	0.046	0.048	0.171
5	Risk strat index 1	0.052	0.068	0.067	0.096
6	employed_lodds	0.045	0.056	0.057	0.13
7	hispanic_percent	0.042	0.044	0.043	0.098
8	urban_lodds	0.039	0.049	0.048	0.09
9	ANEMIA	0.038	0.008	0.005	0.135
10	Risk strat index 2	0.031	0.047	0.048	0.063
11	CCI	0.031	0.026	0.029	0.118
12	vacant_housing	0.028	0.038	0.038	0.052
13	PreOp_Systolic	0.027	0.035	0.033	0.111
14	black_percent	0.025	0.028	0.033	0.093
15	PreOp_Diastolic	0.025	0.036	0.029	0.11
16	PreOp.HR	0.023	0.024	0.029	0.055
17	ed_college	0.022	0.039	0.038	0.092

© 2020 King CR et al. *JAMA Network Open.*

18	white_percent	0.018	0.028	0.024	0.058
19	SEX	0.017	0.016	0.019	0.135
20	Total.	0.017	0.04	0.033	0.085
21	poverty_fraction	0.016	0.026	0.024	0.051
22	ed_hs	0.015	0.032	0.029	0.059
23	CANCER_HX	0.014	0.017	0.019	0.029
24	SMOKING_EVER	0.012	0.007	0.01	0.076
25	RACEBlack	0.011	0.008	0.005	0.057
26	before_screeningTRUE	0.011	0.003	0.005	0.042
27	asian_percent	0.009	0.011	0.01	0.052
28	ASA	0.009	0.007	0.005	0.05
29	FUNCTIONAL_CAPACITY	0.008	0.006	0.005	0.062
30	DM	0.007	0.004	0.005	0.047
31	neval_valid	0.007	0.005	0.01	0.056
32	case_year2013	0.007	0.011	0.01	0.019
33	CHF	0.006	0.006	0.005	0.04
34	CAD_PRIORMI	0.006	0.007	0.005	0.04
35	blank_preop	0.005	0.003	0.005	0.035
36	Surg_TypeUROLOGY	0.004	0.006	0.005	0.033

© 2020 King CR et al. *JAMA Network Open.*

37	GERD	0.004	0.003	0.005	0.026
38	RACEWhite	0.003	0.005	0.005	0.028
39	CKD	0.003	0.007	0.005	0.036
40	Surg_TypeGENERAL	0.003	0.007	0.005	0.032
41	case_year2016	0.003	0.006	0.005	0.031
42	HTN	0.002	0.004	0.005	0.022
43	case_year2015	0.002	0.006	0.005	0.027
44	PreOp.SpO2	0.001	0.003	0.005	0.018

Predicting Delirium

	**Feature**	**Gain**	**Cover**	**Frequency**	**shap**
1	ccs_factor_216	0.144	0.033	0.018	0.221
2	ASA	0.105	0.036	0.026	0.279
3	PreOp.HR	0.074	0.051	0.055	0.174
4	Risk strat index 1	0.059	0.082	0.089	0.104
5	emergency	0.049	0.018	0.011	0.146
6	Risk strat index 2	0.042	0.065	0.059	0.1
7	Age	0.037	0.053	0.048	0.182
8	ccs_factor_34	0.036	0.015	0.007	0.066

© 2020 King CR et al. *JAMA Network Open.*

9	FUNCTIONAL_CAPACITY	0.032	0.018	0.018	0.128
10	vacant_housing	0.026	0.046	0.041	0.122
11	white_percent	0.024	0.035	0.044	0.098
12	WEIGHT	0.023	0.032	0.044	0.048
13	Total.	0.021	0.03	0.041	0.047
14	BMI	0.02	0.037	0.041	0.083
15	ed_less_hs	0.02	0.024	0.033	0.056
16	PreOp_Diastolic	0.019	0.025	0.022	0.08
17	employed_lodds	0.018	0.027	0.033	0.036
18	before_screeningTRUE	0.017	0.012	0.015	0.079
19	black_percent	0.015	0.019	0.022	0.078
20	PreOp.SpO2	0.015	0.016	0.018	0.039
21	PreOp_Systolic	0.015	0.026	0.022	0.06
22	ed_college	0.015	0.026	0.033	0.045
23	urban_lodds	0.013	0.027	0.026	0.065
24	PAP_TypeOTHER	0.013	0.012	0.015	0.065
25	PAP_TypeIPAP	0.012	0.012	0.015	0.069
26	asian_percent	0.012	0.01	0.018	0.024
27	CKD	0.012	0.01	0.007	0.043

© 2020 King CR et al. *JAMA Network Open.*

28	CCI	0.012	0.019	0.015	0.055
29	poverty_fraction	0.011	0.011	0.018	0.029
30	hispanic_percent	0.009	0.012	0.018	0.025
31	ccs_factor_50	0.006	0.004	0.004	0.026
32	ed_hs	0.006	0.012	0.011	0.031
33	ccs_factor_49	0.006	0.01	0.007	0.038
34	ccs_factor_44	0.006	0.019	0.011	0.039
35	RACEWhite	0.005	0.009	0.007	0.034
36	Surg_TypeORTHOPEDIC	0.005	0.012	0.007	0.025
37	case_year2015	0.004	0.008	0.007	0.036
38	neval_valid	0.004	0.003	0.004	0.022
39	CANCER_HX	0.004	0.011	0.007	0.038
40	ccs_factor_176	0.004	0.007	0.004	0.017
41	GERD	0.004	0.009	0.007	0.036
42	DM	0.003	0.008	0.007	0.026
43	SMOKING_EVER	0.003	0.001	0.004	0.013
44	HTN	0.003	0.006	0.004	0.029
45	ccs_factor_158	0.003	0.006	0.004	0.013
46	DVT	0.002	0.007	0.004	0.014

© 2020 King CR et al. *JAMA Network Open.*

47	ccs_factor_222	0.002	0.007	0.004	0.014
48	Surg_TypeUNKNOWN	0.002	0.002	0.004	0.012
49	CHF	0.002	0.004	0.004	0.016
50	CAD	0.002	0.007	0.004	0.022
51	ANEMIA	0.002	0.004	0.004	0.014
52	blank_preop	0.001	0.007	0.004	0.023
53	SEX	0.001	0.001	0.004	0.008

Predicting OSA

	**Feature**	**Gain**	**Cover**	**Frequency**	**shap**
1	WEIGHT	0.245	0.076	0.072	0.32
2	BMI	0.116	0.096	0.094	0.554
3	HTN	0.104	0.014	0.016	0.285
4	Age	0.053	0.031	0.028	0.287
5	before_screeningTRUE	0.05	0.018	0.016	0.297
6	Risk strat index 1	0.037	0.071	0.063	0.151
7	GERD	0.027	0.011	0.016	0.185
8	employed_lodds	0.025	0.045	0.06	0.095
9	Risk strat index 2	0.02	0.048	0.047	0.094

© 2020 King CR et al. *JAMA Network Open.*

10	SEX	0.019	0.029	0.022	0.332
11	PAP_TypeIPAP	0.018	0.016	0.019	0.175
12	hispanic_percent	0.016	0.027	0.031	0.102
13	ed_hs	0.016	0.034	0.041	0.09
14	PreOp_Systolic	0.015	0.035	0.038	0.062
15	PPM_ICD	0.015	0.006	0.003	0.077
16	PreOp.HR	0.014	0.033	0.038	0.087
17	ed_less_hs	0.014	0.025	0.025	0.095
18	PAP_TypeOTHER	0.013	0.011	0.009	0.15
19	PreOp_Diastolic	0.013	0.033	0.034	0.06
20	blank_preop	0.013	0.004	0.003	0.089
21	COPD	0.013	0.015	0.009	0.127
22	asian_percent	0.013	0.033	0.031	0.07
23	white_percent	0.012	0.024	0.028	0.087
24	ed_college	0.012	0.022	0.028	0.07
25	black_percent	0.011	0.029	0.028	0.078
26	urban_lodds	0.01	0.019	0.022	0.091
27	Total.	0.009	0.017	0.022	0.062
28	vacant_housing	0.009	0.022	0.022	0.077

© 2020 King CR et al. *JAMA Network Open.*

29	poverty_fraction	0.009	0.023	0.022	0.087
30	PHTN	0.008	0.01	0.006	0.095
31	Surg_TypeUNKNOWN	0.008	0.002	0.003	0.035
32	PreOp.SpO2	0.008	0.015	0.016	0.066
33	CCI	0.004	0.014	0.013	0.064
34	RACEWhite	0.004	0.007	0.006	0.074
35	DM	0.004	0.007	0.006	0.072
36	ccs_factor_43	0.003	0.009	0.006	0.018
37	SMOKING_EVER	0.003	0.004	0.006	0.06
38	neval_valid	0.003	0.01	0.009	0.034
39	ccs_factor_61	0.002	0.005	0.003	0.036
40	ccs_factor_50	0.002	0.007	0.006	0.045
41	ASA	0.002	0.001	0.003	0.023
42	ccs_factor_216	0.001	0.005	0.003	0.029
43	CKD	0.001	0.004	0.003	0.028
44	CAD_PRIORMI	0.001	0.005	0.003	0.027
45	emergency	0.001	0.005	0.003	0.031
46	case_year2013	0.001	0.005	0.003	0.033
47	FUNCTIONAL_CAPACITY	0.001	0.005	0.003	0.031

© 2020 King CR et al. *JAMA Network Open.*

48	Surg_TypeGENERAL	0.001	0.005	0.003	0.022
49	ANEMIA	0.001	0.005	0.003	0.031
50	CANCER_HX	0.001	0.004	0.003	0.021

© 2020 King CR et al. *JAMA Network Open*.

eAppendix 8. Propensity Score Balance Diagnostics

Empirical cumulative distribution plots for raw sample (left) and OSA propensity score reweighted sample (right) stratified by OSA plus high risk screen status. Y-axis is the fraction of observed data below the threshold, x-axis is a threshold value of the variable of interest. Non-informative confounding variables gives overlapping curves.

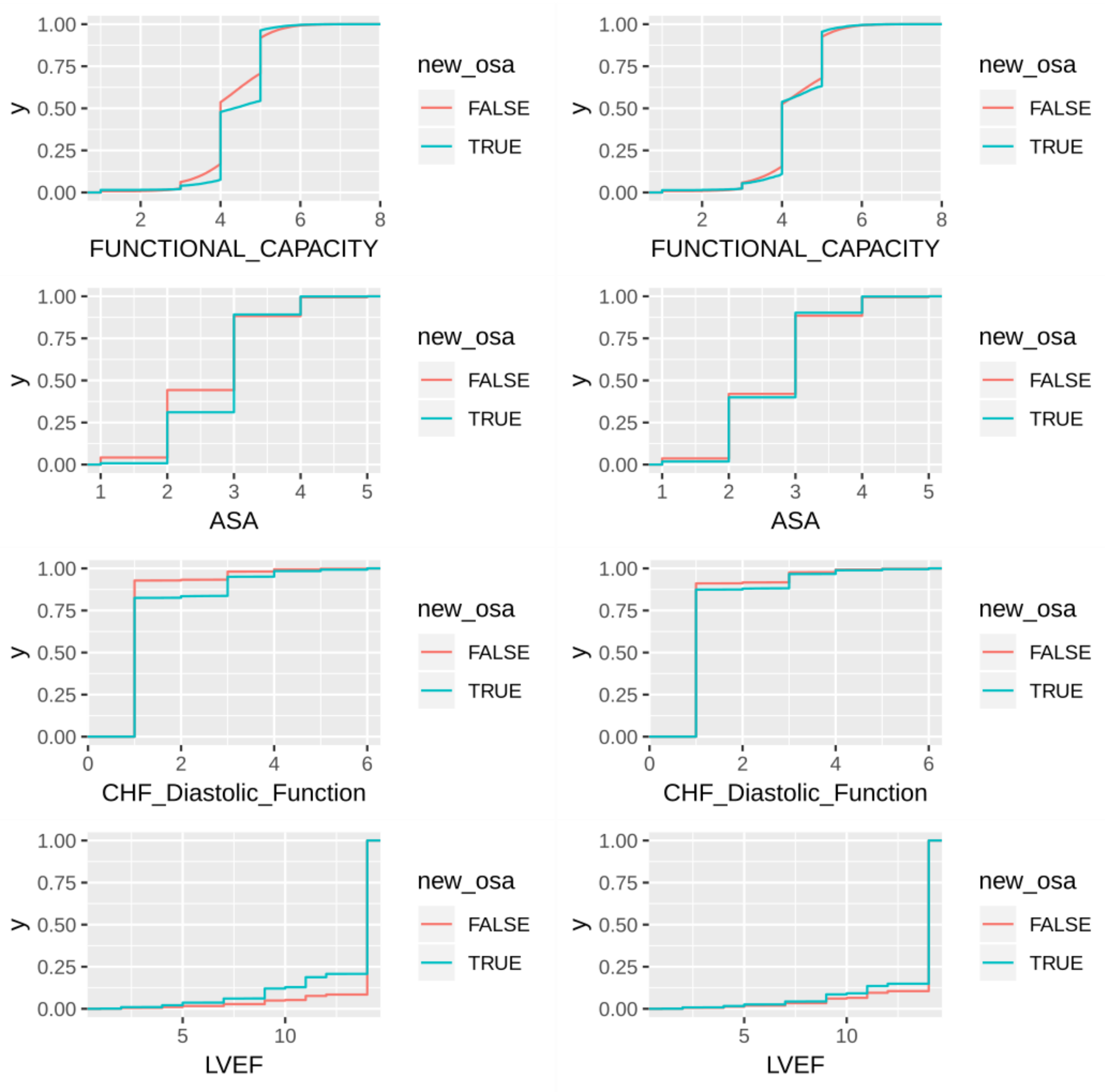

© 2020 King CR et al. *JAMA Network Open.*

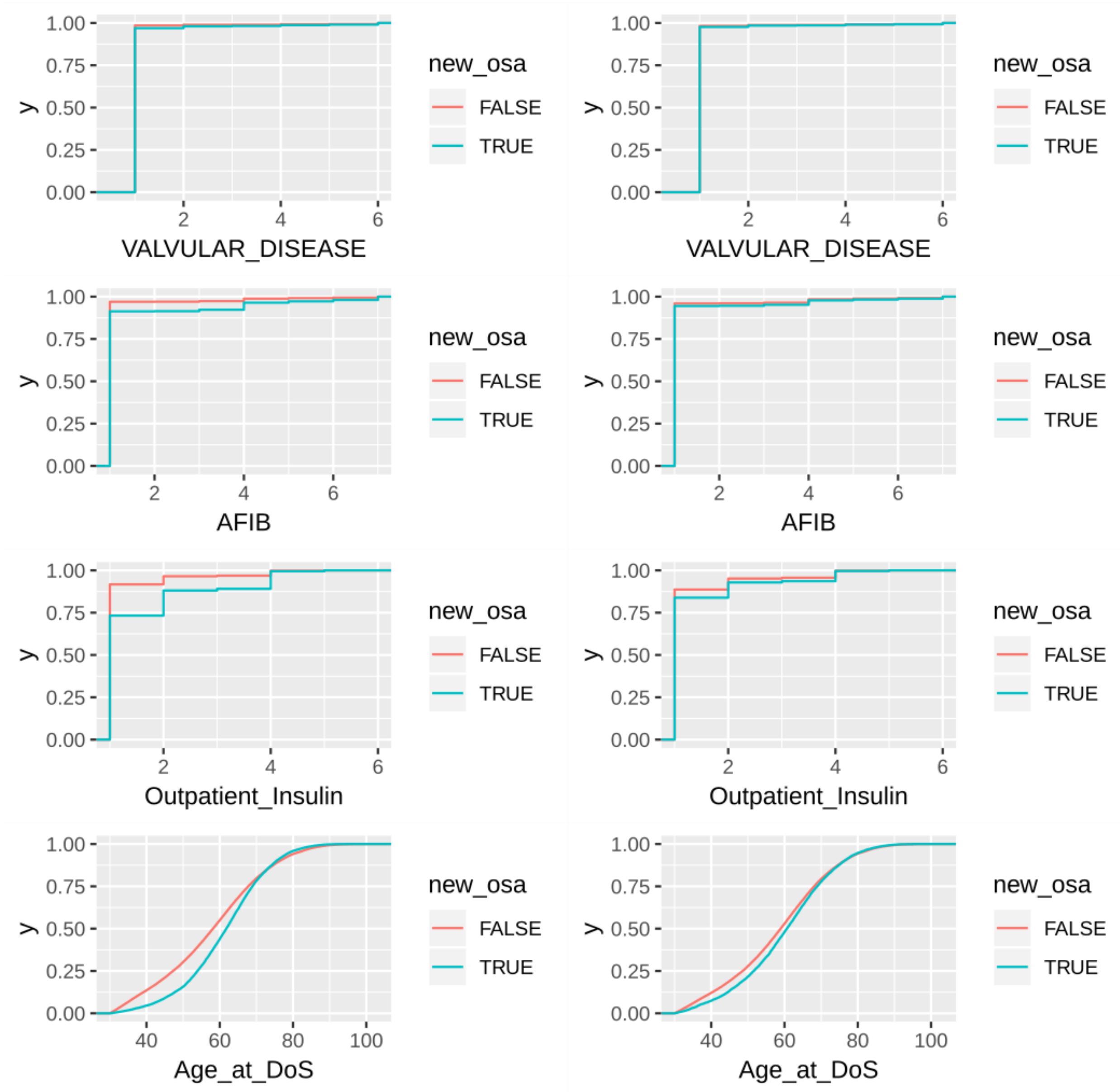

© 2020 King CR et al. *JAMA Network Open.*

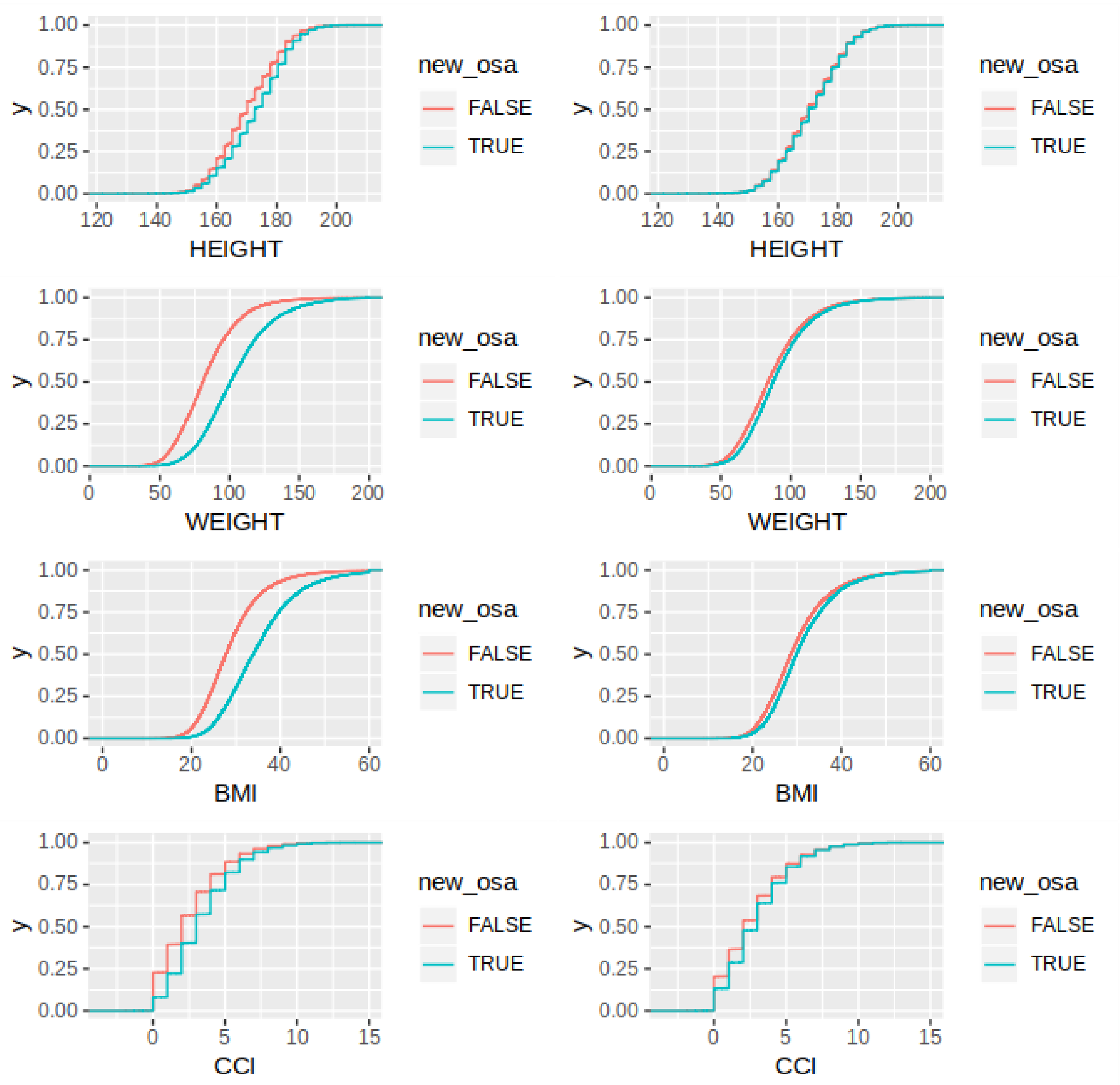

© 2020 King CR et al. *JAMA Network Open.*

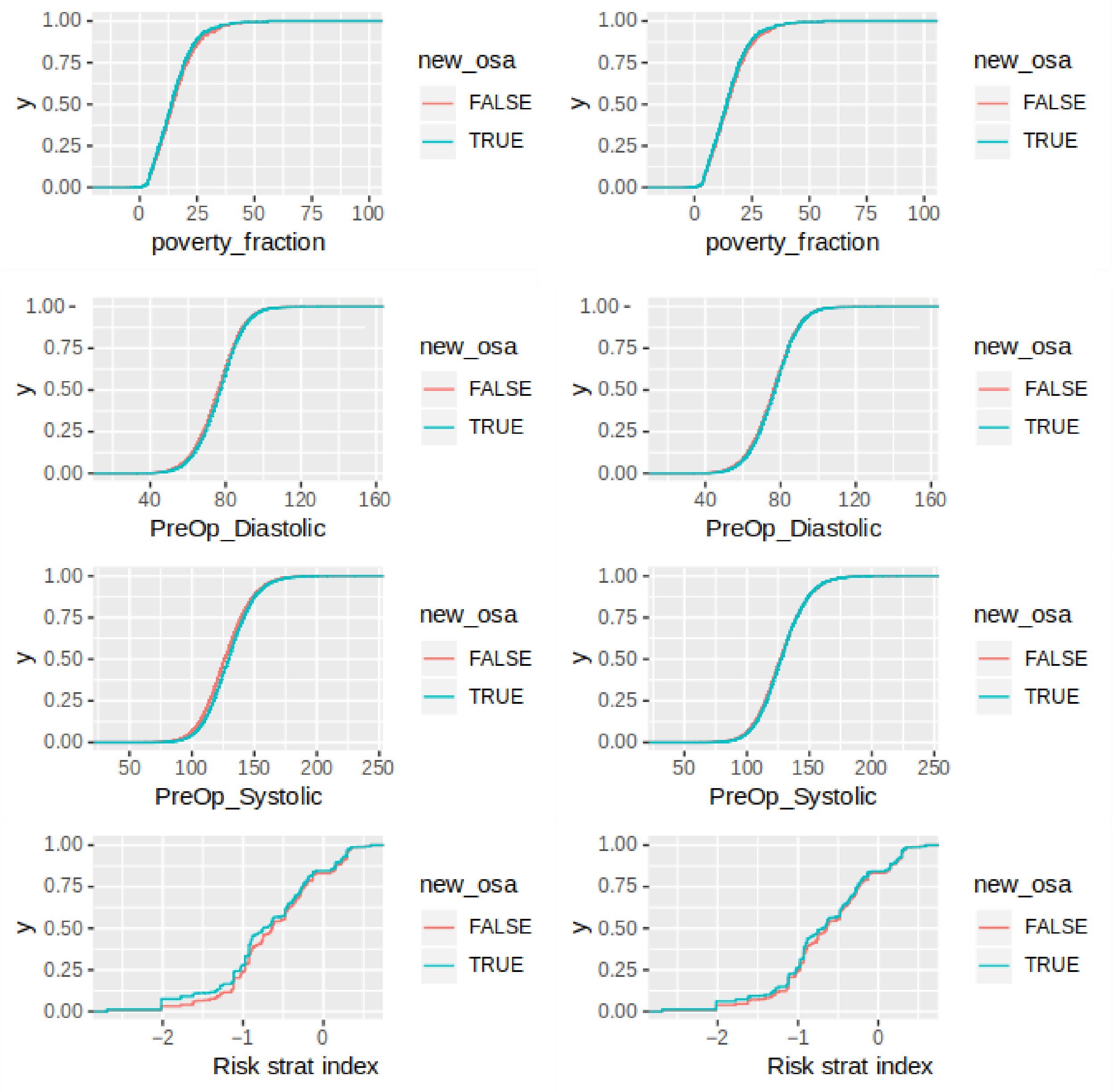

© 2020 King CR et al. *JAMA Network Open.*

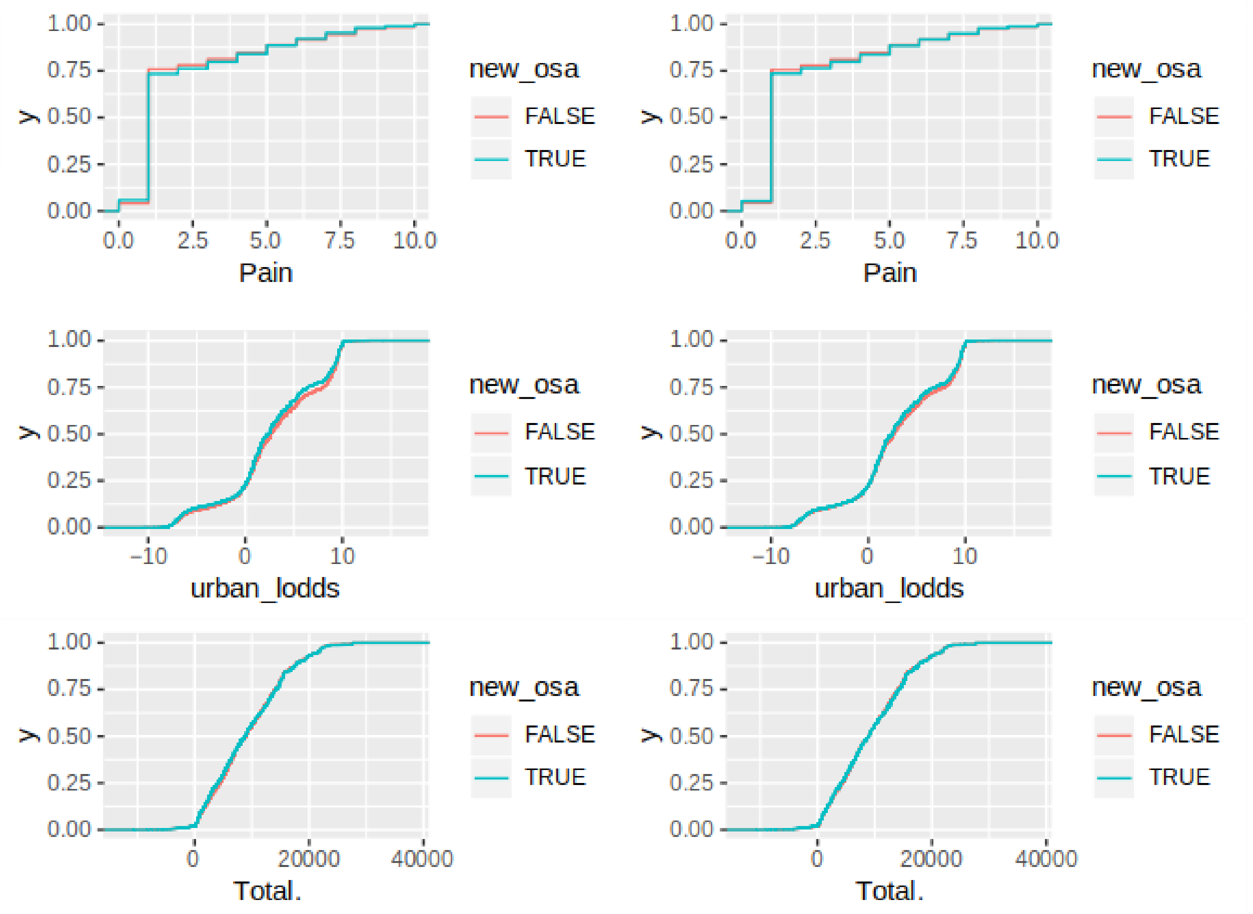

© 2020 King CR et al. *JAMA Network Open.*

eReferences

1. Buuren S van, Groothuis-Oudshoorn K. mice: Multivariate Imputation by Chained Equations in R. *Journal of Statistical Software*. 2011;45(1):1-67. doi:10.18637/jss.v045.i03
2. von Hippel PT. Regression with Missing Ys: An Improved Strategy for Analyzing Multiply Imputed Data. *Sociological Methodology*. 2007;37:83-117. doi:10.1111/j.1467-9531.2007.00180.x
3. Sullivan TR, Salter AB, Ryan P, Lee KJ. Bias and Precision of the "Multiple Imputation, Then Deletion" Method for Dealing With Missing Outcome Data. *Am J Epidemiol*. 2015;182(6):528-534. doi:10.1093/aje/kwv100
4. Leyrat C, Seaman SR, White IR, et al. Propensity score analysis with partially observed covariates: How should multiple imputation be used? *Stat Methods Med Res*. 2019;28(1):3-19. doi:10.1177/0962280217713032
5. Zhou X, Reiter JP. A Note on Bayesian Inference After Multiple Imputation. *The American Statistician*. January 2012. doi:10.1198/tast.2010.09109
6. Si Y, Reiter JP. A Comparison of Posterior Simulation and Inference by Combining Rules for Multiple Imputation. *J Stat Theory Pract*. 2011;5(2):335-347. doi:10/ggbsmq
7. He J, Yalov S, Hahn PR. XBART: Accelerated Bayesian Additive Regression Trees. In: *The 22nd International Conference on Artificial Intelligence and Statistics*. ; 2019:1130-1138.
8. Hahn PR, Carvalho CM, Puelz D, He J. Regularization and Confounding in Linear Regression for Treatment Effect Estimation. *Bayesian Anal*. 2018;13(1):163-182. doi:10.1214/16-BA1044
9. Saarela O, Belzile LR, Stephens DA. A Bayesian view of doubly robust causal inference. *Biometrika*. 2016;103(3):667-681. doi:10.1093/biomet/asw025
10. Hill JL. Bayesian Nonparametric Modeling for Causal Inference. *Journal of Computational and Graphical Statistics*. 2011;20(1):217-240. doi:10.1198/jcgs.2010.08162
11. Hahn PR, Murray JS, Carvalho C. Bayesian regression tree models for causal inference: regularization, confounding, and heterogeneous effects. *arXiv:170609523 [stat]*. June 2017. http://arxiv.org/abs/1706.09523. Accessed March 3, 2018.
12. Dorie V, Hill J, Shalit U, Scott M, Cervone D. Automated versus Do-It-Yourself Methods for Causal Inference: Lessons Learned from a Data Analysis Competition. *Statist Sci*. 2019;34(1):43-68. doi:10.1214/18-STS667
13. Hansen BB, Klopfer SO. Optimal full matching and related designs via network flows. *Journal of computational and Graphical Statistics*. 2006;15(3):609-627. doi:10.1198/106186006X137047
14. Lundberg S, Lee S-I. A Unified Approach to Interpreting Model Predictions. *arXiv:170507874 [cs, stat]*. May 2017. http://arxiv.org/abs/1705.07874. Accessed November 20, 2018.

© 2020 King CR et al. *JAMA Network Open.*
